# Research ReportDiurnal global ocean surface *p*CO_2_ and air–sea CO_2_ flux reconstructed from spaceborne LiDAR data

**DOI:** 10.1093/pnasnexus/pgad432

**Published:** 2023-12-14

**Authors:** Siqi Zhang, Peng Chen, Yongxiang Hu, Zhenhua Zhang, Cédric Jamet, Xiaomei Lu, Davide Dionisi, Delu Pan

**Affiliations:** State Key Laboratory of Satellite Ocean Environment Dynamics, Second Institute of Oceanography, Ministry of Natural Resources, Hangzhou 310012, China; Southern Marine Science and Engineering Guangdong Laboratory (Guangzhou), Nansha District, Guangzhou 511458, China; State Key Laboratory of Tropical Oceanography, South China Sea Institute of Oceanology, Chinese Academy of Sciences, Guangzhou 510301, China; State Key Laboratory of Satellite Ocean Environment Dynamics, Second Institute of Oceanography, Ministry of Natural Resources, Hangzhou 310012, China; Southern Marine Science and Engineering Guangdong Laboratory (Guangzhou), Nansha District, Guangzhou 511458, China; National Aeronautics and Space Administration Langley Research Center, Hampton, VA 23681, USA; State Key Laboratory of Satellite Ocean Environment Dynamics, Second Institute of Oceanography, Ministry of Natural Resources, Hangzhou 310012, China; Southern Marine Science and Engineering Guangdong Laboratory (Guangzhou), Nansha District, Guangzhou 511458, China; Laboratoire d’Océanologie et de Géosciences (LOG), Université Littoral Côte d’Opale, CNRS, Université Lille, 62930 Wimereux, France; National Aeronautics and Space Administration Langley Research Center, Hampton, VA 23681, USA; Institute of Marine Sciences (ISMAR), Italian National Research Council (CNR), Rome - Tor Vergata 700185, Italy; State Key Laboratory of Satellite Ocean Environment Dynamics, Second Institute of Oceanography, Ministry of Natural Resources, Hangzhou 310012, China; Southern Marine Science and Engineering Guangdong Laboratory (Guangzhou), Nansha District, Guangzhou 511458, China; State Key Laboratory of Tropical Oceanography, South China Sea Institute of Oceanology, Chinese Academy of Sciences, Guangzhou 510301, China

**Keywords:** diurnal variation, air–sea CO_2_ flux, CALIPSO, LiDAR, remote sensing

## Abstract

The ocean absorbs a significant amount of carbon dioxide (CO_2_) from the atmosphere, helping regulate Earth's climate. However, our knowledge of ocean CO_2_ sink levels remains limited. This research focused on assessing daily changes in ocean CO_2_ sink levels and air–sea CO_2_ exchange, using a new technique. We used LiDAR technology, which provides continuous measurements during day and night, to estimate global ocean CO_2_ absorption over 23 years. Our model successfully reproduced sea surface partial pressure of CO_2_ data. The results suggest the total amount of CO_2_ absorbed by oceans is higher at night than during the day. This difference arises from a combination of factors like temperatures, winds, photosynthesis, and respiration. Understanding these daily fluctuations can improve predictions of ocean CO_2_ uptake. It may also help explain why current carbon budget calculations are not fully balanced—an issue scientists have grappled with. Overall, this pioneering study highlights the value of LiDAR's unique day–night ocean data coverage. The findings advance knowledge of ocean carbon cycles and their role in climate regulation. They underscore the need to incorporate day–night variability when assessing the ocean's carbon sink capacity.

**Significance Statement**

It is very difficult (probably impossible) to measure ocean surface *p*CO_2_ directly from space. The best proxy of *p*CO_2_ that can be measured from space is likely ocean biological properties (e.g. primary productivity, Chla, etc.) that are very sensitive to pH level and thus dissolved organic carbon (DIC)/*p*CO_2_, together with other measurable physical properties (e.g. temperature, ocean dynamics, etc.). Training the biological and physical properties of *p*CO_2_ from in situ measurements sounds like a very rational approach. Diurnal changes in ocean surface *p*CO_2_ could affect ocean carbon sinks, which is only beginning to be assessed nowadays. This study helps fill this knowledge gap by employing a cutting-edge satellite technology—LiDAR. LiDAR can probe the oceans day and night using laser pulses. We have developed a new model based on a feed-forward neural network incorporating LiDAR data as input to estimate global ocean CO_2_ absorption from 1998 to 2020 at a monthly resolution. For the first time, this approach harnesses unique advantages of LiDAR's day–night ocean observations. The results offer new insights into daily CO_2_ fluctuations and their climate implications. Overall, this work demonstrates the value of an emerging technique for improving climate predictions and informing policy decisions.

## Introduction

The global ocean plays a vital role in mitigating climate change by absorbing atmospheric carbon dioxide (CO_2_), a heat-trapping greenhouse gas. Scientists estimate that oceans currently absorb about 25% of human-generated CO_2_ emissions (1). However, gaps remain in understanding the ocean carbon cycle. Comprehensive global measurements of the surface partial pressure of CO_2_ (*p*CO_2_) are lacking. This hampers efforts to predict ocean CO_2_ uptake, model climate change impacts, and inform policymaking.

Comprehending the onset and progression of ocean acidification necessitates a thorough understanding of the entire cycle of CO_2_ variability (2). Since 1850, the ocean carbon sink has increased in tandem with the exponential rise in anthropogenic emissions, resulting in the marine CO_2_ sink reaching 3.0 ± 0.4 GtC year^−1^ in 2020 (3). However, the carbon budget imbalance, which represents the disparity between the estimated total emissions and the estimated changes in the atmosphere, ocean, and terrestrial biosphere, is approximately 0.1–0.3 GtC year^−1^ (3–5). This imbalance serves as an indicator of incomplete data and our current understanding of the contemporary carbon cycle (4). The long-term variations in the ocean surface *p*CO_2_ act as the principal driving force governing CO_2_ exchange across the air–sea interface (6). Consequently, sea surface *p*CO_2_ observations assume a pivotal role in constraining the global air–sea carbon sink. Regrettably, such knowledge remains limited due to the scarcity of comprehensive global sea surface *p*CO_2_ observations. Recent global estimates still obscure noteworthy interannual and regional fluctuations (7), underscoring the necessity for rigorous quantitative research to meticulously trace the precise evolution of the Earth's carbon budget (8).

Benefiting from the growing abundance of global ocean biogeochemical remote sensing data and the continuous advancements in remote sensing retrieval methods, the estimation of long-term series of sea surface *p*CO_2_ is progressively achieving higher levels of accuracy. A variety of data-interpolation approaches provide estimates of the surface ocean *p*CO_2_ field (7) such as statistical interpolation, linear and nonlinear regression, or model-based regressions or tuning (9). Artificial neural networks have succeeded in filling the spatial and temporal gaps. Different artificial neural networks have been widely used to reconstruct sea surface *p*CO_2_ in the global ocean (10–18). In contrast, the existing products usually present monthly fields with a 1° × 1° spatial resolution, with large errors occurring in Antarctica and the Arctic due to the problem with input remote sensing data (18).

While average states and seasonal variations in ocean CO_2_ system variables have received relatively comprehensive characterization, the assessment of diurnal changes is only in its infant stages (19, 20). Previous observation-based studies have explored the diurnal variability of surface ocean *p*CO_2_ and reported extreme diurnal amplitudes of 187 ± 85 μatm in the open ocean (20), 5–25 μatm in the open-ocean Sargasso Sea (21), 8 and 15 μatm in the tropics and <5 μatm in the subtropics (22). Higher diurnal amplitudes for *p*CO_2_ (100–500 μatm) have been recorded near benthic ecosystems such as coral reefs (20, 23, 24), kelp forests (25), and seagrasses (26), particularly in shallow waters, where the percentage of benthic biomass is enhanced (27, 28). Diel *p*CO_2_ variations provide essential insights into the dynamic processes of the carbon cycle and affect ocean acidification (29). Diurnal *p*CO_2_ variations also influence marine biological productivity, which, in turn, can impact the entire marine food web (30). Moreover, changes in ocean temperature and circulation can alter CO_2_ uptake and release processes, potentially leading to positive or negative feedback loops that either amplify or mitigate climate change (31, 32). In summary, understanding of oceanic processes and aids in the formulation of effective environmental policies and conservation measures. It provides valuable data for policymakers and researchers working toward sustainable ocean management and mitigating the impacts of climate change. However, there is no study on global diurnal ocean surface *p*CO_2_ and air–sea CO_2_ flux (C-flux) reconstruction considering both diurnal thermodynamic influences and biochemical effects. Moreover, traditional passive remote sensing data failed to provide information during nighttime, adding an additional challenge to addressing this critical knowledge gap.

During the last two decades, the Cloud-Aerosol Lidar with Orthogonal Polarization (CALIOP) has been the primary instrument on the cloud-aerosol LiDAR flown aboard the Cloud-Aerosol Lidar and Infrared Pathfinder Satellite Observations (CALIPSO) platform, providing day-and-night measurements comparable with those of mature passive remote sensing radiometry since June 2006 (33–38). CALIOP is a polar orbiting sensor that conducts daytime and nighttime (approximately 13:40 and 01:40 local time, respectively) near-nadir backscattering measurements along its orbit track at a sampling frequency that is equivalent to every 330 m on the ground. Recent studies have demonstrated that CALIOP also collects information about the ocean at both the global scale and in specific regions: Hu et al. (39) estimated wind speed and wave slope variance on a global scale; Hu et al. (40) suggested that the higher spatial resolution (70 m) wind from CALIOP could help reduce uncertainties in air–sea exchange; Behrenfeld et al. (34) provided global maps of particulate backscattering observed from CALIOP; Lu et al. (35) showed the global sea surface chlorophyll-a concentration (Chla) and particulate organic carbon concentrations estimated from CALIOP; and Behrenfeld et al. (37, 41) measured the annual cycles of phytoplankton biomass in polar regions and studied global satellite-observed daily vertical migrations of ocean animals. Compared with traditional satellite ocean color remote sensing, CALIOP measurements could provide new observations of seawater phytoplankton properties for both day and night, globally and in polar regions, to improve the understanding of global phytoplankton primary productivity and carbon stocks/fluxes (42–45). Despite the likely importance of these changes, the consequences of increased diel chemistry variation for marine organisms and ecosystem processes remain almost entirely unexplored.

Overall, satellites have enabled remote sensing of key ocean properties related to CO_2_ absorption like temperature, plankton levels, and wind speeds. However, traditional satellites only provide data during daylight hours. Nighttime changes in CO_2_ linked to winds, temperatures, and biology remain poorly characterized. This study helps fill this knowledge gap by employing a cutting-edge satellite technology—LiDAR. LiDAR can probe the oceans day and night using laser pulses. Within this frame, our objectives were 2-fold: firstly, to explore the potential application of CALIOP diurnal Chla and wind speed products in studying the global air–sea carbon cycle and secondly, to examine the long-term diurnal *p*CO_2_ and air–sea C-flux using multisource satellite products, which include LiDAR products. To achieve this, we have proposed a novel feed-forward neural network model with LiDAR data inputs (FNN-LID) to estimate global long-term diurnal *p*CO_2_ and C-flux. The best proxy for *p*CO_2_ that can be measured from space is likely ocean biological properties (e.g. primary productivity, Chla, etc.) that are very sensitive to pH level and thus DIC/*p*CO_2_, together with other measurable physical properties (e.g. temperature, ocean dynamics, etc.). By employing this model, we were able to establish a nonlinear and continuous relationship between climatology *p*CO_2_ and various independent environmental predictors, such as Chla, sea surface temperature (SST), and others, derived from remote sensing data. The study period covered from January 1998 to December 2020 at a monthly 1°× 1° resolution. Notably, this research represents the pioneering effort to incorporate spaceborne LiDAR diurnal data in constructing global diurnal ocean surface *p*CO_2_ and C-flux. To assess the performance and accuracy of our model, we conducted a comprehensive comparison with eight other estimates based on the fugacity of CO_2_ (*f*CO_2_) from Global Carbon Budget 2020 (GCP2020). In particular, we compared the CALIOP-retrieved Chla against consistent observations obtained from biogeochemical Argo profiling floats (46). Similarly, we validated the wind speed data from buoys located across the equatorial and subequatorial oceans. Subsequently, we were able to estimate the long-term series of global ocean surface *p*CO_2_ and C-flux. In addition, we summarize the developed approach and the main results and provide recommendations for future biogeochemical studies using LiDAR active remote sensing measurements. These findings shed light on crucial aspects of the air–sea carbon cycle and contribute to our understanding of the Earth's carbon dynamics on a global scale. In Materials and methods section, we describe the CALIOP data, in situ observation data and other used remote sensing environmental data. Then, we introduce the adopted and refined procedures to retrieve the diurnal Chla and wind speed from CALIOP and the ocean surface *p*CO_2_ and air–sea C-flux estimated based on the FNN-LID method.

## Results

### FNN-LID validation

**Fig. 1.**
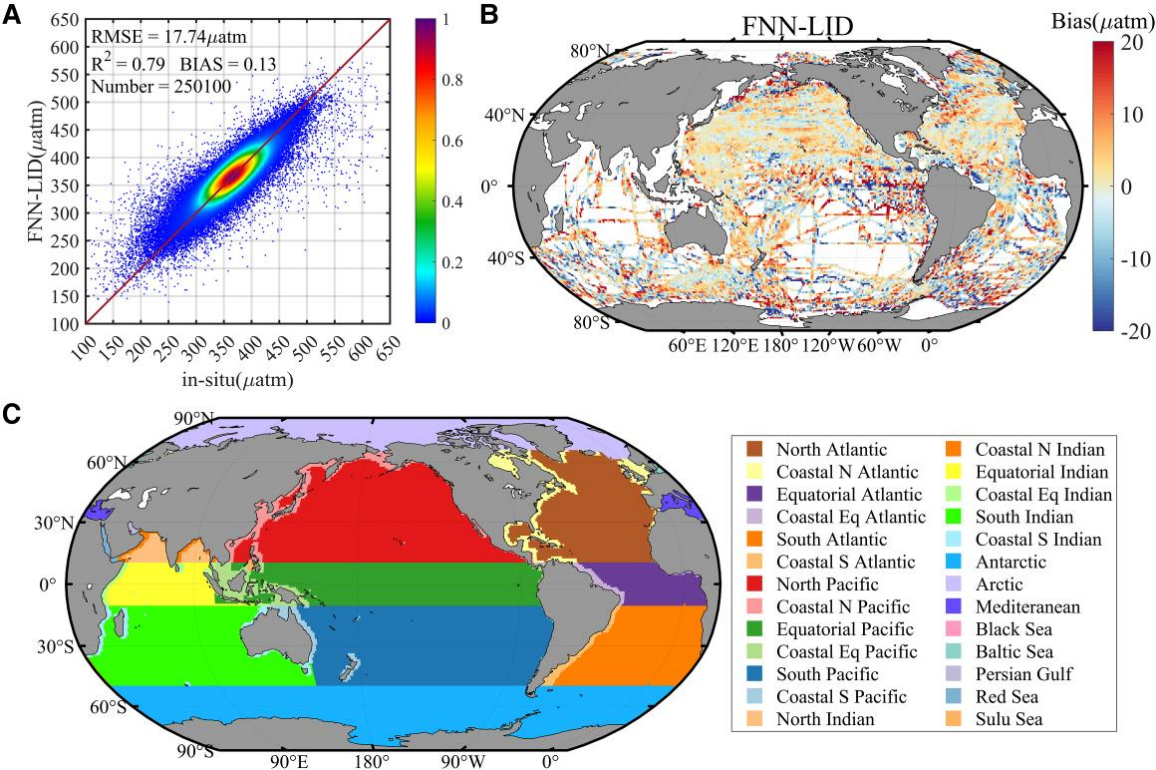
A) Observation of global surface ocean *p*CO_2_ (μatm) and FNN-LID estimates. The color code represents the density of occurrences. The line is the 1:1 line. B) Map of the mean of the differences between FNN-LID *p*CO_2_ and SOCAT gridded dataset (47). The surface ocean *p*CO_2_ is colocalized with observations and the bias is calculated. Then, this bias is summed in time and the average. C) The masks of 26 global oceans from World Ocean Database, NOAA to validate the surface ocean *p*CO_2_ in different regional oceans (available at https://www.nodc.noaa.gov/OC5/WOD/wod_masks.html).

In this study, we validated the global estimates of FNN-LID *p*CO_2_ data against both observed data and widely used reconstructed datasets. The observed data utilized in the validation process consisted of unmodeled gridded data from the SOCATv2022 Gridded Dataset (47). The results revealed exceptional long-term agreement with a remarkable *r*^2^ value of 0.79, a low root mean square error (RMSE) of 17.74 μatm, and an almost negligible overall bias of 0.05 μatm considering 250,100 matched gridded observations (Fig. 1A). For statistics, please refer to the SI Appendix. This strong global fit, encompassing both open ocean and coastal regions, was consistently observed for each individual year, demonstrating high levels of consistency, with *r*^2^ values ranging from 0.73 to 0.90, RMSE ranging from 15 to 21 μatm and annual bias remaining within a narrow range of ±2 μatm (SI Appendix, Table S1). The reanalysis datasets included *p*CO_2_ data reconstructed using FNN from the Copernicus Marine Environment Monitoring Service (CMEMS) and *p*CO_2_ data reconstructed using SOM-FNN from Biogeochemistry and Pollutant Dynamics (IBP) (16, 17, 48). The RMSE for the CMEMS global estimates ranged from 17 to 26 μatm, while the IBP estimates exhibited an RMSE ranging from 9 to 13 μatm over the open ocean and 4 to 32.9 µatm over coastal seas. Globally, the FNN-LID model demonstrated an excellent fit with the gridded *p*CO_2_ data from SOCATv2022, showcasing accuracy levels similar to those of other recent models in the mean monthly result. These findings further support the reliability and effectiveness of the FNN-LID approach for global *p*CO_2_ estimation.

The right panel of Fig. 1B presents the temporal mean residuals when comparing them to the SOCAT map in each pixel. Overall, the bias was minimal and exhibited a random distribution in space in most midlatitude open oceans (e.g. North Pacific and North Atlantic). However, it tended to increase in regions sparse or limited observation data (e.g. Indian Ocean and Southern Ocean) as well as in areas characterized by extremely high or low *p*CO_2_ (e.g. East Equatorial Pacific and Labrador Sea). Regions with high spatial variability exhibited relatively poorer model fits, whereas the FNN-LID model demonstrated notably good performance in capturing the dynamics of most less variable open-ocean regions.

To validate the surface ocean *p*CO_2_ across different regional oceans and distinguish the coastal from the open ocean, we utilized the global ocean masks from the World Ocean Database, NOAA (available at https://www.nodc.noaa.gov/OC5/WOD/wod_masks.html). Further details regarding the regional (open and coastal) division are provided in Fig. 1C. The North Pacific stands out as a region with high data coverage and a rapid increase in data availability since 2003 was observed. The corresponding RMSEs for the North Pacific, the Equatorial Pacific, and the South Pacific were below 18 µatm, and *r*^2^ values ranged between 0.76 and 0.84. Although the RMSEs were generally low across the Pacific, skillful data reconstruction over the coastal North Pacific regions posed challenges. RMSEs were generally below 20 µatm, with a larger RMSE of 39.52 µatm obtained for the Coastal North Pacific. The large model–data mismatch along the Pacific continental shelves reflects the poor reconstruction of *p*CO_2_ over regions under the influence of upwelling systems (e.g. Kuroshio Current), large river discharges (e.g. Bering Sea shelf), and the bottlenecks of gulfs or bays (e.g. South China Sea). As discussed in Hales et al. (49), the carbon cycling in the Coastal Pacific is subject to complex dynamics, exhibiting high spatial and temporal variability driven by multiple physical and biogeochemical drivers. These intricate spatial and temporal changes occur within the 1°×1° grid contribute to the large RMSE for the reconstructed product. On the other hand, a comparison between the gridded observed data and evaluation data across the six subregions of the Atlantic reveals small mean model–data differences, leading to high reconstruction skill in the Atlantic basin (SI Appendix, Table S2). The coastal Atlantic regions performed particularly well in terms of reconstruction, accounting for over 29% of the total coastal data. Mean RMSE is below 25 µatm, and, with the exception of the coastal (*r*^2^ = 0.71). Corresponding RMSDs are 14.33 (North Atlantic), 15.09 (Equatorial Atlantic), and 13.73 µatm (South Atlantic), with *r*^2^ values ranging approximately between 0.66 and 0.77.

In polar regions, FNN-LID *p*CO_2_ exhibits high quality in the Arctic (RMSE of 25.3 µatm), which is comparable with the recent SOCAT-based global *p*CO_2_ fields (RMSEs of 26.7–32 µatm) and provides a more accurate estimate of the polar continental shelf in winter (50). This advantage stems from the fact that FNN-LID uses CALIPSO which has advantages over traditional passive remote sensing (the Sea-viewing Wide Field-of-view Sensor [SeaWiFS] or Moderate Resolution Imaging Spectroradiometer [MODIS]) in distinguishing clouds and sea ice (51). Therefore, active satellite LiDAR input enables continuous observation of the winter polar nighttime period, obtaining high-coverage polar inputs (52). Thus, we constructed a LiDAR-based *p*CO_2_ with far greater coverage than previous products.

In conclusion, the residual analysis demonstrates that the global FNN-LID method successfully meets most criteria for a robust fit, with no significant evidence of hidden biases. Notably, the estimates do not show any substantial degradation concerning data density, be it in temporal or spatial dimensions. Regions with pronounced spatial or temporal variability exhibit relatively weaker fits, whereas the method excels in accurately estimating *p*CO_2_ for most open-ocean regions characterized by lower variability.

### Interannual *p*CO_2_ and air–sea flux

**Fig. 2.**
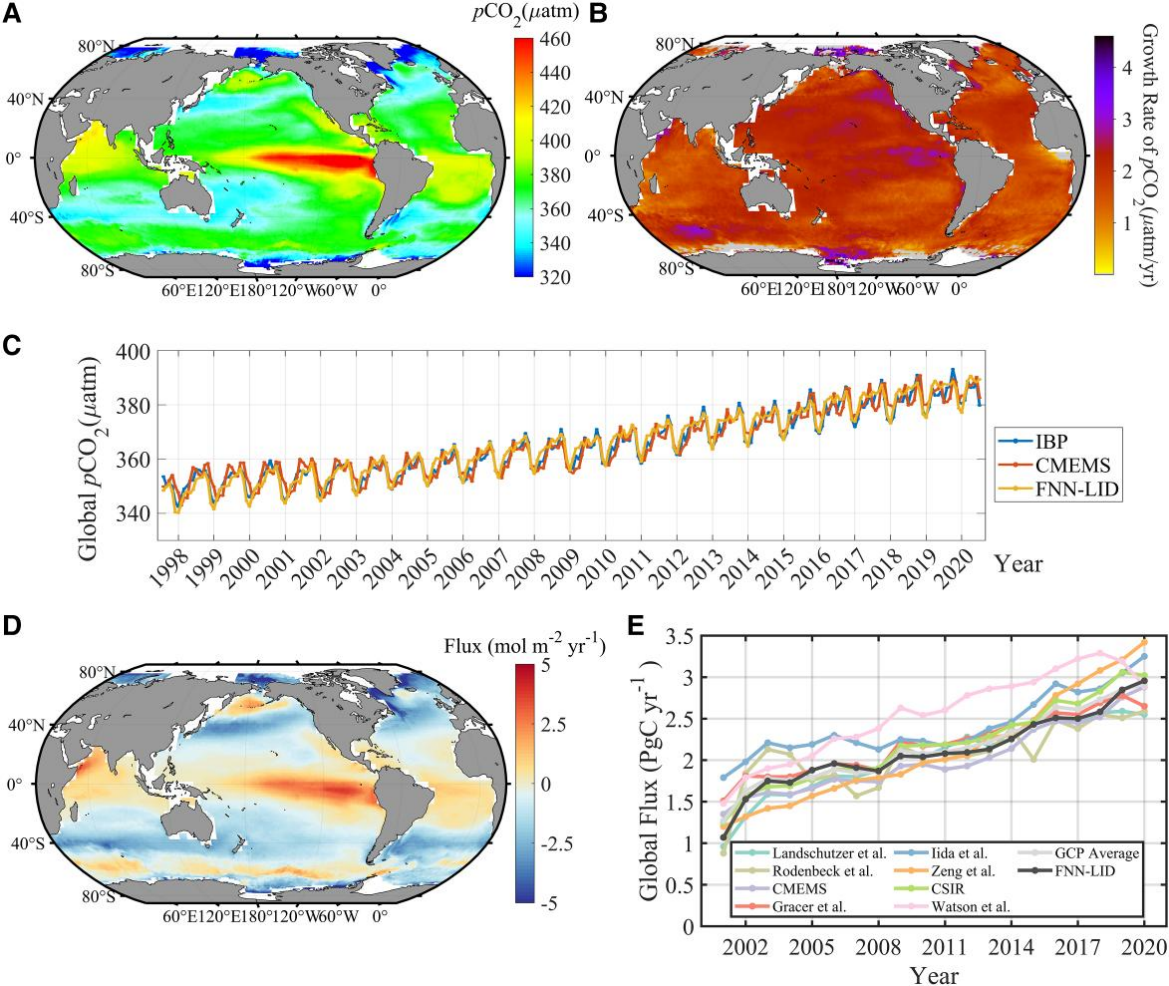
The distribution of A) climatological annual mean *p*CO_2_ in µatm and B) mean growth rate in µatm year^−1^. C) Temporal evolution of the global *p*CO_2_ (in µatm) from blue FNN-LID, IBP, CMEMS, and estimates during 1998–2020. D) Map of the long-term mean annual net air–sea flux for CO_2_ (mol C m^−2^ year^−1^) for 1998–2020. Red–yellow areas indicate that the ocean is a source for atmospheric CO_2_, and blue–indigo areas indicate that the ocean is a CO_2_ sink. E) Comparison of the global carbon budget in this study (black line) with the 8 *f*CO_2_-based estimates from GCP2020 from 2001 through 2020. All values are in GtC year^−1^, and positive flux densities indicate CO_2_ uptake by the ocean.

Utilizing the FNN-LID methodology, we successfully obtained the climatological surface ocean *p*CO_2_ during 1998–2020 (Fig. 2A). The highest long-term mean sea surface *p*CO_2_ climatology values identified by our FNN-LID method occurred in the equatorial Pacific, which was associated with the strong upwelling of deep water with naturally rich dissolved inorganic carbon. Similarly, elevated *p*CO_2_ levels were also identified in the northeastern Indian Ocean, the low-latitude South Atlantic and the western basin of the Bering Sea (Fig. 2A). Conversely, regions with the lowest sea surface *p*CO_2_ values were detected in the high latitudes (>70°) of the Atlantic, along the strong western boundary current (Gulf Stream and Kuroshio Current), and in the subtropical bands of the Southern Hemisphere, a combination of both the drawdown of DIC by biological activity and the low-temperature effect on solubility (12). Furthermore, we conducted an analysis of the interannual increasing rate for each 1°×1° pixel, as illustrated in Fig. 2B. The results indicate a significant upward trend, with an average increasing rate of approximately 1.8 (±0.7 µatm year^−1^), among which the Ross Sea, East Siberian Sea, and Beaufort Sea growth was set to be sharply over 4 µatm year^−1^. These findings were corroborated by multiple models, and to validate our approach, we compared the global monthly average *p*CO_2_ data with CMEMS and the Institute of IBP over the period from 1998 to 2020, as presented in Fig. 2C. The comparison of sea surface *p*CO_2_ from the three mapping approaches displayed minor variations within the range of ±3 µatm. On a global scale, the three products gave extremely similar results for a long time series. However, it is worth noting that the IBP method exhibited a slight overestimation of observed *p*CO_2_, particularly during the period 1998–2003, with deviations of up to 3 µatm. On the other hand, the discrepancy between the CMEMS output and our approach fluctuated within the range of ±2 µatm, with an increase in amplitude of up to −4 µatm from November 2020.

Figure 2D displays the long-term mean C-flux density in mol C m^−2^ year^−1^, providing insights into the average annual net atmosphere–ocean C-flux. The results indicated CO_2_ release predominantly at low latitudes and uptake at high latitudes, with the exception of the Bering Sea, which acts as a source of CO_2_ to the atmosphere. The detailed calculation steps for the C-flux are available in SI Appendix. Remarkably, we identified significant sink areas in the transition zone between the subtropical gyre and subpolar waters (i.e. 30°–50° latitudes). This phenomenon primarily arises due to the cooling of subtropical warm waters and secondarily to the biological drawdown of *p*CO_2_ in nutrient-rich subpolar waters. Moreover, areas with high wind speeds were found to enhance the CO_2_ sink capability of ocean waters. The eastern equatorial Pacific, northwestern Arabian Sea and western Bering Sea were the most intense CO_2_ source areas (red regions in Fig. 2D). The equatorial Pacific, particularly in the eastern upwelling area, was highly supersaturated with regard to atmospheric *p*CO_2_ and constituted a strong source region for air. Additionally, the tropical Atlantic, Pacific, and Indian Oceans and the subarctic region, also exhibited significant source characteristics for CO_2_.

The regional distribution of C-flux in the world's oceans has been a subject of scientific interest, and recent research highlights the significance of previously overlooked areas, such as the Arctic. In the past, limited data coverage of input data in previous studies has taken up approximately 15+% of the global ocean CO_2_ uptake flux (SI Appendix, Table S3). Similarly, the Atlantic Ocean covers roughly 23% of the ocean area and accounts for approximately 31+% of the global ocean CO_2_ uptake. In contrast, the Pacific Ocean accounts for only 25% of the global ocean CO_2_ uptake, whereas it occupies 47% of the global ocean area. The lower CO_2_ sink is formed by the juxtaposition of the intense CO_2_ source in the equatorial Pacific with a strong seasonal source in the Bering Sea. The dynamics of this region are further influenced by El Niño events, involving decreased upwelling of carbon in the equatorial Pacific due to a weakening of the trade winds. This region will become a weaker sink of CO_2_ or will become near neutral if the El Niño event is strong (53). The total CO_2_ flux was scaled by the ratio of the total ocean area covered by the respective product to the total ocean area (361.9 × 10^6^ km^2^) (54, 55). Furthermore, it should be noted that the ocean sink we discussed here does not contain carbon from river inputs to the ocean, which are approximately 0.61 GtC year^−1^ (the average of 0.45 ± 0.18 GtC year^−1^ by (56) and 0.78 ± 0.41 GtC year^−1^ by (57)). Figure 2E illustrates the result that our FNN-LID-based global contemporary air–sea C-flux exhibited a modest level of year-to-year variability from 2001 to 2020, with a minimum carbon uptake of −1.10 ± 0.32 PgC year^−1^ in 2001 and reached a maximum uptake of −2.7 ± 0.47 PgC year^−1^ in 2020 and with a standard deviation of the de-seasonalized and detrended (to separate the effect of short-term trends) monthly fluxes of (±0.09 PgC year^−1^). We showed a similar result in both annual estimation and interannual variation to the eight results mentioned in GCP2020 (3).

### The global diurnal ocean surface *p*CO_2_ and flux

**Fig. 3.**
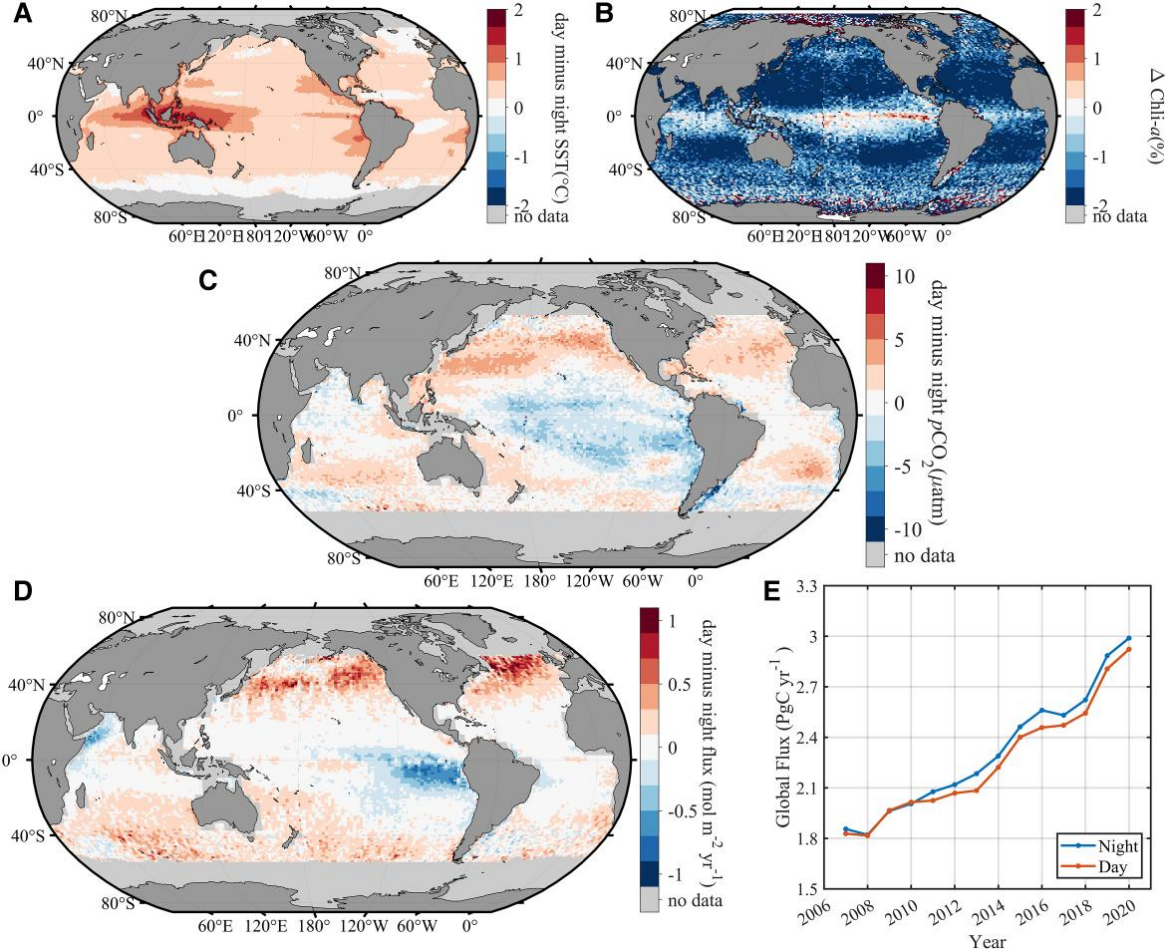
The annual mean difference of day and night A) SST, B) ΔChla, and C) *p*CO_2_, among ΔChla is shown as the normalized difference ratio. C) The map of global diurnal air–sea C-flux difference (day minus night) in mol m^−2^ year^−1^; D) comparison of the CO_2_ flux showing the budget values of ocean at nighttime and at daytime in GtC year^−1^.

The above sections have substantiated the availability of LiDAR products for their potential application in *p*CO_2_ studies. Furthermore, we have demonstrated diurnal *p*CO_2_ based on CALIPSO satellite products and multisource diurnal products. We used the diurnal SST and Chla to reconstruct the diurnal *p*CO_2_ fields (henceforth referred to as the FNN-LID) by applying the FNN-LID, and the climatological diurnal differences in SST and Chla are shown in Fig. 3A and B. Fields of diurnal and nocturnal transfer velocity were computed using wind speed observations from CALIPSO. These fields were then used to compute the monthly mean fields of the daytime and nighttime air–sea CO_2_ fluxes. Due to the resolution of the 1°×1° grid and the lack of diurnal temperature data at high latitudes, our credible diurnal *p*CO_2_ data would cover open oceans between latitudes of 60°S-60°N, as shown in Fig. 3C. Next, we specifically describe the influencing factors and potential causes of diurnal variation in seawater *p*CO_2_.

Temperature is a pivotal parameter in carbonate systems because it affects *p*CO_2_ in isochemical conditions (∂ln *p*CO_2_/∂*T*) with a rate of +4.23% °C^−1^ (58). The acute daily cycle is influenced by solar radiation, wind speed, the optical attenuation coefficient of the water, and mixing from the wave motions (59). This diurnally varying SST rather than daily averaged SST affects air–sea fluxes (60). Figure 3A indicates that the mean amplitude in *Δ* SST for the entire ocean is 0.47°C (±0.26°C), with the largest *Δ*SSTs exceeding 1.7°C in Indonesia and affecting 0.01% of the surface.

However, the thermodynamic effect was dampened by primary production during the day, as shown in Fig. 3B. Light affects phytoplankton activity, leading to diel periodicity in cell division and cellular properties (61–66). The Chla difference ratio (Eq. 1) yields a negative value in almost the entire open sea (Fig. 3B):


(1) $$\Delta \mathrm{Chla}=\frac{\mathrm{Chl}a_{\mathrm{day}}-\mathrm{Chl}a_{\mathrm{night}}}{\mathrm{Chl}a_{\mathrm{day}}}\times 100\text{\%}$$

In contrast, the marginal sea Chla concentrations were higher during the day, such as in the northwestern Arabian Sea, Gulf of California, and the Arafura Sea. The same pattern was found in equatorial regions, especially the eastern equatorial Pacific. The global annual mean diurnal variation pattern of ΔChla agreed well with the study by Behrenfeld et al. (41). Synchronized cell division during nighttime has been confirmed for many phytoplankton groups, resulting in a decrease in cell size during this period of the day, which may cause diel changes (41, 61, 63, 65, 66). Regardless of the ecological reasons behind the existence of diel rhythms in Chla, their consequences in day and night are different. In addition, these diel variations have been neglected in all previous research regarding the dependence of passive remote sensing upon solar radiation.

The annual mean diurnal variations in *p*CO_2_ (d*p*CO_2_ = day–night) are displayed in Fig. 3C. The variations are a comprehensive consequence of diurnal SST variations, leading to an increase (due to deep-water upwelling and sea–air CO_2_ exchange) or decrease (due to photosynthesis and sea–air CO_2_ exchange) in the total CO_2_ concentration. The spatial distribution of these variations is unique, showing positive d*p*CO_2_ in the subequatorial and midlatitude regions (higher diurnal *p*CO_2_), while it was negative in the equatorial Pacific and Southeast Pacific (higher nocturnal *p*CO_2_). The lowest d*p*CO_2_ (>10 μatm) was exhibited in Patagonia. Diurnal variations were small in the tropics and typically <5 μatm. The strongest diurnal variations occurred in the northern subtropics and midlatitudes, where we estimated that *p*CO_2_ was typically lowered by between 3 and 7 μatm during nighttime compared with daytime. In the Southern Hemisphere, diurnal variations were lower, between 3 and 6 μatm. Negative d*p*CO_2_ appeared in upwelling regions, such as in the western Arabian Sea. Many previous studies have shown that an increase in seawater temperature will cause changes in the carbonate composition of seawater, resulting in a larger *p*CO_2_. In summary, diurnal *p*CO_2_ change is not a simple change due to temperature or Chla but is rather caused by a combination of seawater inorganic carbonate systems and a series of complex organic biochemical processes.

As for diurnal flux, there are differences in the magnitude of the fluxes between day and night (Fig. 3D). The diurnal flux variations are mostly positive, indicating that the evasion in source regions is increased during the day, whereas the invasion in sink regions is decreased. Diurnal flux variations are strongest in the southern midlatitudes where the day—night flux difference is ∼1 mol m^−2^ year^−1^. However, in the tropics and West Arabian Sea diurnal variations are negative and sometimes even <0.5 mol m^−2^ year^−1^ (e.g. at 100°W). Combined with the climatology air–sea C-flux, the regions that be deemed as carbon sources are more likely to release more CO_2_ at night than during the day. Figure 3E shows the long-term series global flux, revealing that the nocturnal sink is higher than the diurnal one. Furthermore, if we keep using the diurnal uptake as the whole daily sinks, it will cause underestimation by between −0.005 and 0.052 GtC year^−1^ (0.026 GtC year^−1^ on average). This may be helpful to answer the question of where part (approximately 10–30%) of the total carbon budget imbalance (about 0.1–0.3 GtC year^−1^) comes from, over the last decade.

Diurnal *p*CO_2_ plays a crucial role in unraveling the complexities of the carbon cycle, ocean acidification, biological productivity, climate change feedbacks, and environmental health. Jury et al. (29) showed that under global change diel seawater chemistry variation increases (dramatically in some cases) and that various ecosystem feedbacks can substantially modify changes in both the average chemistry and diel chemistry variation. Delille et al. (67) studied diel fluctuations of *p*CO_2_ and DIC inside and outside a giant kelp bed and suggested that understanding the physical and biological processes regulating *p*CO_2_ dynamics facilitates tracking the seasonal evolution of primary production. A workshop on the potential impacts of ocean acidification on marine ecosystems and fisheries indicated the precise control of diel CO_2_ cycling was considered highly valuable in studying the impact of ocean acidification (30). However, diurnal change in ocean surface *p*CO_2_ could affect ocean sink, which is only beginning to be assessed nowadays. This study on diurnal variation may be helpful in answering the question where some of the global carbon budget imbalance over the past decade comes from. Its importance extends to various scientific disciplines, helping shape our understanding of the Earth's interconnected systems and informing strategies to safeguard marine ecosystems and global environmental stability.

## Discussion

### Global seasonal amplitude

The seasonal variations in sea surface *p*CO_2_, as observed at the Hawaiian Ocean time-series station (68) (1.5 ± 1.8 and 0.2 ± 1.8 μatm per decade) and at the Hydrostation “S”/Bermuda Atlantic time-series study site (69–71) (1.5 ± 1.1 μatm per decade) showed a significantly increased in recent decades. Meanwhile, model-based projections supported this result, especially in the Southern, Pacific, and North Atlantic Oceans (2, 72, 73).

Based on the FNN-LID methods, we reconstructed changes in global ocean *p*CO_2_ on a monthly basis with a spatial resolution of 1° × 1° for the period 2001 to 2020. To assess seasonal differences, we calculated winter averages in the Northern Hemisphere as the mean of December, January, and February and the summer averages as the mean of June, July, and August; and the reverse was applied for the Southern Hemisphere. The seasonal differences in *p*CO_2_ were computed here as summer averages minus winter averages. This approximation slightly underestimated seasonal oscillations in the equatorial region, where the highest value may not occur in the summer due to seasonal changes in solar radiation.

Analyzing the mean result over 5-year intervals, we observed that the seasonal differences in surface *p*CO_2_ increased in the Northern Hemisphere, with the winter-to-summer differences becoming more positive in low latitudes (equatorward of ∼40°) and more negative in high latitudes (poleward of ∼40°) (SI Appendix, Fig. S1A–D). Similar spatial patterns were noted in the Southern Hemisphere with a slightly increasing summer-minus-winter difference, except in the South Pacific (SI Appendix, Fig. S1E and F). The change in sign over 40° latitude corresponded to a 6-month phase shift in the seasonal peak of *p*CO_2_ being 6 months out of phase between these two bands. In high latitudes, the seasonal cycle had a maximum in summer, resulting in a negative seasonal difference. In contrast, the seasonal cycle in low latitudes exhibited a peak in winter, leading to a positive summer-minus-winter difference in *p*CO_2_.

Although some residual interannual variability persisted in the strength of the seasonal *p*CO_2_ difference, it is essential to emphasize the explicit positive trend in northern extratropical regions, with an average rate of 2.1 ± 0.6 μatm per decade.

### Accuracy of diurnal air–sea C-flux

Errors in the reconstruction of the global diurnal *p*CO_2_ arise from both the FNN-LID methods and the input data. Unlike the monthly average product, here, our input data were newly added, including diurnal SST, sea surface Chla, wind speeds, and atmospheric *p*CO_2_.

The daily and monthly night SSTs were handled from longwave infrared (LWIR) SST products, a 30+ year record of space-based measurements of LWIR SST from polar orbiting satellites. The current LWIR algorithm is applicable for both daytime and nighttime observations and is based on a modified version of the nonlinear SST algorithm developed by Walton et al. (74), most recently described in Kilpatrick et al. (75). The SST measured by MODIS and VIIRS infrared radiometers is commonly referred to as the skin temperature of the ocean and not the body of water below, as measured by in situ thermometers (76). The thermal skin layer of the ocean is <1 mm thick and cooler than the underlying water (77, 78). For the uncertainty validation for the ESA analysis dataset, the spread of uncertainties in the products ranged from 0.05 to 1.5 K, and the agreement between the theoretical and measured RMSE values was excellent across the full range of uncertainties (79).

For Chla, the global distribution of ocean subsurface backscatter was estimated from the CALIOP level-1 data product, retrieving a diurnal Chla concentration with an accuracy that is (at least as good as) comparable with that obtained from MODIS (35), which can help us better understand the different optical properties of various water masses and facilitate the quantification of global ocean carbon stocks (80). In our analysis, we used MODIS Chla rather than GlobColour Chla, due to the merged product is merging different errors (such as seasonal bias) and uncertainties and the result is not as robust as using one satellite with the same time period as CALIOP (81). The comparison between the independent validation MODIS dataset and the daytime Chla-LID used in this study is shown in SI Appendix, Fig. S2A with an RMSE of 0.85 μg/L and an *r*^2^ value of 0.75. To estimate the accuracy of the nocturnal data, we also compared our products with BIO-Argo within 1° × 1° and 6 h. Although there is a difference between the Chla-LID and BIO-Argo Chla, the bias is insensitive to whether the measurement is taken during the daytime or nighttime, as shown in SI Appendix, Fig. S2B. Although the comparison statistics results for the BIO-Argo measurement were lower than the former, our day and night data maintained a similar estimation ability, providing high-quality nighttime data that could not be retrieved from MODIS. Roesler et al. (82) found that Argo-Chla can be biased by a factor of 2. Additionally, other biological impacts, such as zooplankton diurnal changes, may explain the bias between Argo and CALIOP.

The diurnal wind speed utilized in our study was retrieved from CALIPSO. To validate the neural network approach, we applied CALIPSO measurements for the entire dataset from 2008. The retrieval results were unbiased, with an average wind speed difference between CALIPSO and AMSR-E of approximately −0.32 m/s (SI Appendix, Fig. S2C). Meanwhile, we selected measured buoy data from the National Data Buoy Center, PIPATA, RAMA, and TAO/TRITON as the standards to verify the wind speed. The comparison indicated that the CALIPSO wind speeds may be underestimated (by 1–2 m/s) when the wind speed is <8 m/s in tropical regions (SI Appendix, Fig. S2D); Nevertheless, the CALIPSO data remains globally applicable, particularly for capturing instantaneous wind speeds. Due to the gradient of wind speed with latitude, although the wind speed model we have established has a good inversion capability on a global scale, but some errors may occur in specific regions (e.g. underestimation of low wind speeds in low latitudes). The relationship between mean square wave slope (LiDAR “wind speed”) and air–sea turbulence exchange speed is independent of stability, but the relation between the true wind speed (and thus microwave-based wind measurements, which is a simple empirical fit of buoy-measured wind) and turbulence exchange speed is stability dependent. LiDAR is actually measuring mean square slopes (or turbulence exchange speed) directly and thus its “wind speed” is the correct wind speed for neutral stability. It is a little bit trickier for microwave (radar or radiometer) measurements, since the microwave wavelength (cm) is too close to the wavelength of capillary-gravity waves (cm too). Nighttime mean square slopes (which LiDAR measures directly) might be larger than the daytime ones even if the wind speeds of day and night are the same. So, LiDAR is actually measuring the diurnal change of the turbulence exchange speed. And the diurnal change in turbulence exchange may be underestimated if we use wind derived from microwave measurements.

### Effect of the diurnal air–sea C-flux variation

**Fig. 4.**
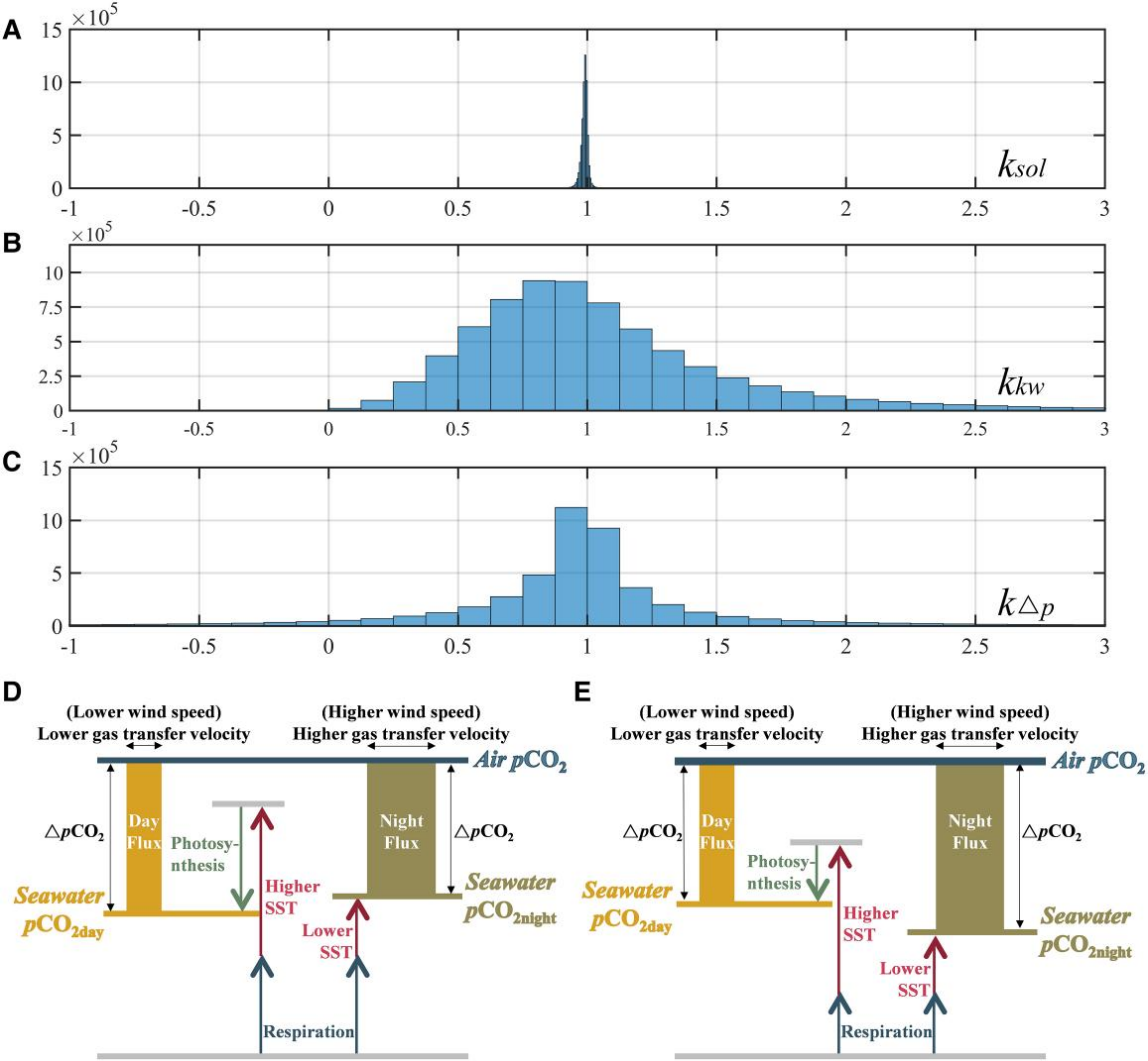
Histograms of diurnal ratios for each parameter: A) *k*_sol_, B) *k_kw_*, and C) *k*_Δ*p*_ involved in CO_2_ flux calculations. Schematic representation of the comprehensive effects on diurnal C-fluxes in two different situations: D) biological effect larger than thermodynamic effect and E) biological effect lower than thermodynamic effect. Colorful arrows represent the effect of different influences on *p*CO_2_, and black ones for the results of synthetic effects. The area of the cylinder represents the amount of CO_2_ flux, and the height of the lines represents the value of *p*CO_2_.

We used the diurnal *p*CO_2_ fields of the FNN-LID and the fields of diurnal *x*CO_2_ and wind speed to estimate the monthly mean fields of daytime and nighttime air–sea C-flux. The calculation of air–sea C-flux appeared in the form of multiplication in this study. Therefore, we will discuss the impact of different mechanisms on C-flux in terms of day–night ratios below:


(2) $$\frac{\mathrm{flu}x_{\mathrm{day}}}{\mathrm{flu}x_{\mathrm{night}}}=\frac{\mathrm{so}l_{\mathrm{day}}}{\mathrm{so}l_{\mathrm{night}}}\times \frac{kw_{\mathrm{day}}}{kw_{\mathrm{night}}}\times \frac{\Delta p_{\mathrm{day}}}{\Delta p_{\mathrm{night}}}=k_{\mathrm{sol}}\times k_{kw}\times k_{\Delta p}$$

(3) $$k_{\mathrm{sol}}=\frac{\mathrm{so}l_{\mathrm{day}}}{\mathrm{so}l_{\mathrm{night}}}$$

(4) $$k_{kw}=\frac{kw_{\mathrm{day}}}{kw_{\mathrm{night}}}$$

(5) $$k_{\Delta p}=\frac{\Delta p_{\mathrm{day}}}{\Delta p_{\mathrm{night}}}$$

where $\mathrm{flu}x_{\mathrm{day}}$, $\mathrm{so}l_{\mathrm{day}}$, $kw_{\mathrm{day}}$, and $\Delta p_{\mathrm{day}}$ represent the air–sea CO_2_ flux, the solubility of CO_2_ in seawater, the gas transfer velocity, and the difference in CO_2_ partial pressure between the ocean surface and the atmosphere during the day; while $\mathrm{flu}x_{\mathrm{night}}$, $\mathrm{so}l_{\mathrm{night}}$, $kw_{\mathrm{night}}$, and $\Delta p_{\mathrm{night}}$ for the night. Furthermore, $k_{\mathrm{sol}}$, $k_{kw}$, and $k_{\Delta p}$ characterize the ratios of the different components in the day and night, respectively (Fig. 4A–C). When this ratio is <1, it means that the parameter is lower during the day, and the opposite means that the larger value occurs during the day.

In previous studies, the combined effect of $k_{\mathrm{sol}}$ was found to be negligible, as it typically remains relatively stable (22). During the analysis, we calculated the $k_{\mathrm{sol}}$ for solubility, which was approximately 0.9903 (±0.0091) during 2007–2020 (Fig. 4A). This result indicates that solubility rarely exhibited lower values during the day compared with nighttime; however, the difference was so minimal that it could be safely disregarded.

Following that, we examined the impacts of both the gas transport rate and the air–sea partial pressure difference on the diurnal flux variation, quantified by $k_{\Delta p}$ and $k_{kw}$, respectively. The diurnal partial pressure difference is the only parameter that can yield negative values in the flux estimation. However, only 7% of them were negative, which indicated that diurnal variation did not change the source‒sink properties of the oceans in most cases. However, the absolute average value of $k_{\Delta p}$ was approximately 0.98, ranging from 0.76 to 1.10. The distribution of $k_{\Delta p}$ demonstrated that the partial pressure difference influenced the magnitude of the flux, but this effect was relatively neutral and did not drive the flux to be higher at night than during the day.

The air–sea gas transfer velocity and the square of wind speed were positively correlated with diurnal wind speed. As shown in Fig. 4B, the value of $k_{kw}$ was approximately 0.86 (±0.61), meaning that the larger nighttime wind speed caused a larger gas transfer rate at night. The ratio of $k_{kw}/|k_{\Delta p}|$ ranged from 0.8 to 5, implying that the contribution of wind speed and partial pressure difference to the effect of diurnal flux difference was approximately 7:1. In conclusion, the difference in diurnal air–sea C-flux was mainly controlled by the gas exchange rate affected by the wind speed, while the partial pressure difference determined the diffusion direction of air–sea CO_2_.

Figure 3 reveals that the nocturnal C-flux is larger than during the daytime, influenced by a combination of diffusion, and biological factors, including photosynthesis and respiration. The powerful thermodynamic influence on seawater *p*CO_2_ is evident, as higher daytime temperatures causing an impact on the carbonate balance and solubility, increasing more in the daytime. However, the biological effect is precisely the opposite, indicating higher nocturnal *p*CO_2_ due to the respiration. These two effects determine the direction of diurnal *p*CO_2_ changes. When thermodynamic photosynthesis is powerful, high biomass usually causes obvious respiration at night, resulting in the *p*CO_2_ is larger during the night (Fig. 3A), and vice versa (Fig. 3B). The spatial distribution map of diurnal *p*CO_2_ is shown in Fig. 3C, where both higher nighttime *p*CO_2_ and diurnal *p*CO_2_ are present. Despite the differences in diurnal *p*CO_2_, ocean *p*CO_2_ is lower than atmospheric *p*CO_2_ globally, resulting in seawater being a sink for the atmosphere regardless of day and night. Additionally, the C-flux is affected by diffusion, which makes the wind speed determine the gas exchange rate. As a result, higher wind speeds at night accelerate the absorption of atmospheric CO_2_ at night (Fig. 4A and B). The air–sea flux represents a dynamic steady state that depends on both *p*CO_2_ and the air–sea turbulence exchange velocity. Colder surface water at night over a clear sky due to long wave radiation can help push more CO_2_ into water in two ways: (i) colder water allows more gases in the water than warmer water; and (ii) mixed layer is more unstable at night and thus helps increase turbulence exchange velocity, when the drop in surface water temperature leads to heavier water at the surface. Furthermore, in the combined of all effects, the gas transfer rate flux has the greatest impact as mentioned in Fig. 4B. Hence, the global annual average C-flux is larger than at daytime under the multieffects.

In this study, we constructed a new FNN-LID method including CALIOP data for global *p*CO_2_ and formed a dataset of long time-series variations in *p*CO_2_ and air–sea CO_2_ fluxes during 1998–2020, as well as diurnal products from 2007 to 2020. Instead of normal passive remote sensing products, we added CALIOP diurnal Chla and wind speed as the input data to build the FNN-LID approach. This is the first time LiDAR has been used in research on the air–sea carbon cycle. CALIOP can provide not only a larger coverage, including the polar regions, but also a clearer understanding of the global diurnal *p*CO_2_ variations.

Globally, our FNN-LID demonstrated excellent fitting capabilities with the gridded *p*CO_2_ data from in situ SOCATv2022, achieving an *r*^2^ value of 0.79 and a negligible overall bias of 0.05 μatm over the entire time period from 1998 to 2020. These results showcased a similar spatiotemporal distribution and accuracy to those of other recent models in the mean monthly result. In addition, we found that *p*CO_2_ exhibited different spatial characteristics during the day and at night by analyzing the FNN-LID results. It exhibited a unique spatial distribution, showing positive d*p*CO_2_ in the subequatorial and midlatitude regions (higher diurnal *p*CO_2_), while it was negative in the equatorial Pacific and southeast Pacific (higher nocturnal *p*CO_2_), and the lowest d*p*CO_2_ (more than 10 μatm) was exhibited in Patagonia. The nocturnal CO_2_ sink was higher than the diurnal sink, leasing to a potential underestimation between −0.005 and 0.052 GtC year^−1^ if the diurnal uptake was used as the whole daily sink. This may be helpful in answering the question of where part of the carbon budget imbalance (approximately 0.1 GtC year^−1^) came from over the last decade. Meanwhile, the contribution of wind speed and partial pressure difference to the effect of diurnal flux difference is approximately 7:1. The partial pressure difference played a crucial role in determining the diffusion direction of air–sea CO_2_ exchange.

This model's application has extended the use of satellite remote sensing data in polar research and diurnal variation studies. Moving forward, with the accumulation of remote sensing data, we will further extend the study of medium time scale carbonate systems. Additionally, we will explore the utilization of higher spatial and temporal resolution data to analyze carbonate systems in coastal and nearshore ecosystems, further enhancing our understanding of these vital marine environments.

## Materials and methods

### Observation data

The gridded monthly *p*CO_2_ data were sourced from the gridded SOCATv2022 observational database (available at https://www.socat.info/) (83). SOCATv2022 provides comprehensive global sea surface fugacity of CO_2_ (*f*CO_2_) data from moorings, ships, and drifters spanning the period from 1970 to 2020. Furthermore, wind speed data from the tropical moored buoys, along with *b*_bp_ and Chla products from an array of Bio-Argo floats, were utilized to validate the accuracy of CALIOP Chla, and the wind speed. Refer to SI Appendix for preprocessing of observation measurements in this study.

### CALIOP measurements

The active remote sensing data came from CALIOP, developed by NASA (available at http://orca.science.oregonstate.edu/lidar.data.php), including CALIPSO level 1B V4.10 data products, LiDAR level 2 Cloud, Aerosol, and Merged Layer V4.20 products. The measured signal is corrected for after-pulse and polarization crosstalk effects (84) before further processed. Following these corrections, $b_{\mathrm{bp}}$ can be calculated from the vertical–parallel ratio (34, 85). Finally, Chla can be estimated based on the relational formula of $b_{\mathrm{bp}}$ and chlorophyll-a concentration *C*.

Ocean surface roughness increases with higher wind speeds, resulting in fewer photons reaching the LiDAR receiver. Thus, the LiDAR backscatter signal is proportionally related to the probability that the surface of the capillary-gravity waves is perpendicular to the line-of-sight of the laser beam (86). In this study, wind speed data were retrieved from LiDAR data from Version 4 CALIPSO level 1 data in combination with collocated ocean surface wind speed data from AMSR-E measurements (87). Refer to SI Appendix for further information on the method and the fitting neural network (SI Appendix, Table S4).

### Satellite and reanalyzed environmental datasets

As shown in SI Appendix, Table S5, our predictors included biological, chemical, and physical variables commonly associated with variations in *p*CO_2_ (13, 17, 88). These predictors comprised SST, sea surface salinity, sea surface height, mixed-layer depth, Chla, and atmospheric CO_2_ mole fraction (*x*CO_2_). It is noteworthy to mention that the Chla used in this study is based on optical measurements rather than analyzed Chla data. In addition to the predictors listed in this table, *p*CO_2_ climatology (89), normalized latitude, and longitude were also utilized as predictors for the reconstruction. Furthermore, the calculation of the C-flux requires additional datasets, including atmospheric pressure, and 10 m wind speed at the sea surface. The original data were distributed after interpolation on 1° latitude by 1° longitude cells. For reconstructing the diurnal *p*CO_2_ field and calculating the air–sea flux, we used all data capable of diurnal spatiotemporal resolution, which encompassed Chla, wind speed, temperature, barometric pressure, and atmospheric CO_2_ concentration. In diurnal research, these data were considered to have the same spatial resolution and began in 2007. See SI Appendix for information on the satellite and reanalyzed environmental datasets used in this study.

### Reconstruction datasets

In the validation of the results, we also employed several widely used *p*CO_2_ reconstruction datasets to corroborate our findings, including *p*CO_2_ data reconstructed using FNN from CMEMS and *p*CO_2_ data reconstructed using SOM-FNN from IBP. These datasets share the same global monthly long-term time series (from 1998 to 2020) at a resolution of 1°×1°.

### FNN-LID model

It is very difficult (probably impossible, or hopeless) to measure ocean surface *p*CO_2_ directly from space. Ocean surface *p*CO_2_ is often constructed based on global ocean biogeochemical models and data reconstruction methods with satellite remote sensing environmental data, SI Appendix, Fig. S3D. In this study, we adopted a novel feed-forward neural network methods including CALIOP data to reconstruct the diurnal *p*CO_2_ for the period from January 1998 to December 2020 at a monthly resolution of 1° × 1°. The two-part method utilized here established nonlinear relationships between *p*CO_2_ and a set of independent environmental predictors. In the first part, we derived a nonlinear and continuous relationship between climatology *p*CO_2_, and the independent environmental predictors based on an FNN method. For the second part, we used the gridded SOCATv2022 observational database as the target.

On this basis, we updated the input data for the diurnal CALIPSO surface sea Chla, diurnal *x*CO_2_ from ECMWF and the diurnal SST product from MODIS during 2007–2020. Hence, we reconstructed the day and night sea surface partial pressure of CO_2_ for the period from January 2007 to December 2021 at a monthly resolution of 1°×1°. For further details on the FNN-LID model, refer to SI Appendix.

## Supplementary Material

pgad432_Supplementary_Data

## Data Availability

All LiDAR data are publicly available at https://zenodo.org/record/7047301. All *p*CO_2_ and C-flux data are included in Supplementary material.
