# Supplementary material for: Research ReportDiurnal global ocean surface pCO2 and air–sea CO2 flux reconstructed from spaceborne LiDAR data
Source: PNAS Nexus. 2023 Dec 14;3(1):pgad432. doi: 10.1093/pnasnexus/pgad432 (PMC10748481; doi:10.1093/pnasnexus/pgad432)
Supplement: pgad432_Supplementary_Data [file pgad432_supplementary_data.zip › PNASNEXUS-PNASNEXUS-2022-01143RR-s01.docx]

**Supporting Information for**

Diurnal Global Ocean Surface *p*CO_2_ and Air-sea Carbon Flux Reconstructed from Spaceborne LiDAR Data

Siqi Zhang, Peng Chen, Yongxiang Hu, Zhenhua Zhang, Cédric Jamet, Xiaomei Lu, Davide Dionisi, and Delu Pan

Peng Chen

Email: chenp[@sio.org.cn](mailto:xxxxx@xxxx.xxx)

**This PDF file includes:**

Supplementary text

Figures S1 to S3

Tables S1 to S5

SI References

Supplemental Materials

**SOCATv2022 *f*CO_2_.** The gridded monthly *p*CO_2_ data were provided by the gridded SOCATv2022 observational database (available at https://www.socat.info/) (1), as shown in *SI Appendix*, Fig. S3*A*. SOCATv2022 represents the global sea surface fugacity of CO_2_ (*f*CO_2_) from moorings, ships, and drifters over the period from 1970 to 2020. To ensure satisfying spatial and temporal data coverage, we limited the reconstruction to the period from 2001 to 2020, which represented approximately 92.53% of the database. The gridded *f*CO_2_ estimates were converted to *p*CO_2_ using the formulation (see, e.g., (2)).

$p\mathrm{CO}_{2}=f\mathrm{CO}_{2}\cdotⅇxp\times{(p\frac{(B+2\delta)}{RT} )}^{-1}$  (1)

where $p\mathrm{CO}_{2}$ and $f\mathrm{CO}_{2}$ are in micro-atmospheres (µatm), $p$ is the total atmospheric surface pressure (Pa), $B$ and $\delta$ are viral coefficients (3), $R$ is the gas constant, and $T$ is the absolute temperature. The National Centers for Environmental Prediction (NCEP) monthly mean sea-level pressure was used for $p$ (4).

**Bio-Argo b_bp_ and Chl-*a*.** The b_bp_ and Chl-*a* product from the array of Bio-Argo floats has been deployed in global areas since 2011(available at http://www.coriolis.eu.org/Data-Products/Data-Delivery/Data-selection), and each float acquires 0–250-m vertical profiles of photosynthetically available radiation at three wavelengths (380, 412, and 490 nm) (5, 6). During 2011–2020, more than 26.5 million profiles were observed, and the day and night measurements allowed us to validate the accuracy of CALIOP Chl-*a*, and the Argo Chl-*a* data averaged to 22.5 m depth (*SI Appendix*, Fig. S3*B*).

**Buoys wind speed.** We used data from tropical moored buoys operated by the TAO (7), TRITON (8), PIRATA (9), and RAMA (10) projects, as well as buoys operated by the National Data Buoy Center (11). The data form these buoys served as a comparison and validation source for CALIOP wind speed, providing hourly measurements of vector winds averaged over a short period (2 to 10 min) at various open oceans (*SI Appendix*, Fig. S3*C*). Additionally, following the procedure described in Liu and Tang (1996), we converted the buoy wind measurements to 10-m neutral stability winds, as CALIPSO wind would be converted to neutral stability wind due to the way the satellite sensors were calibrated. Fig. 13c displays a map of buoy locations with valid data for an example month, December 2016 (12). The tropical Pacific and Indian Oceans show good coverage, as do the coastal regions surrounding North America.

**CALIOP Chl-*a*.** The active remote sensing data came from CALIOP developed by NASA (available at http://orca.science.oregonstate.edu/LiDAR_nature_2019.php). It includes CALIPSO Level 1B V4.10 data products (Kim et al., 2018), LiDAR Level 2 Cloud, Aerosol, and Merged Layer V4.20 products. The CALIOP LiDAR is an active sensor producing simultaneous laser pulses with dual polarization at 532 nm. The measured signal is corrected for after-pulse and polarization crosstalk effects (13) before being processed. The corrected signal can be calculated as follows(14):

$\beta^{'}\left( z \right)=\left[ F \right]^{-1}\beta(z)$ (2)

$\beta_{\parallel,\perp}=\frac{\beta_{\parallel,m}}{1-CT}$ (3)

$\beta_{\perp,c}=\beta_{\perp,m}-CT\times\beta_{\parallel,c}$ (4)

where $\beta^{'}\left( z \right)$ is the corrected backscattered signal, $\beta(z)$ is the output of the receiver, $\left[ F \right]$ is the matrix form of the transient function, CT is the Crosstalk of polarization beam splitters, and $\beta_{\parallel,c}$ and $\beta_{\perp,c}$ are the corrected parallel and perpendicular signals, respectively. After correcting the effects of transient response and crosstalk, b_bp_ can be calculated from the vertical-parallel ratio (15, 16):

$b_{bp}\left( 532 \right)=\frac{2K_{d}\beta_{w+}}{0.32\times{0.98}^{2}}\frac{1+\delta_{p}}{\delta_{p}}\approx\frac{2K_{d}\beta_{w+}}{0.32\times{0.98}^{2}}\frac{1}{2K_{d}}\approx\frac{\beta_{w+}}{0.32\times{0.98}^{2}}$ (5)

where $K_{d}$ is the ocean downwelling diffuse attenuation coefficient, $\beta_{W+}$ is the subsurface column-integrated backscatter of the perpendicular component, and $\delta_{p}$ is the particulate depolarization ratio. Finally, Chl-*a* can be estimated based on the relational formula of $b_{bp}$ and chlorophyll-a concentration $C$, and the formula can be written in the following form (17):

$C= {log}_{0.17} \frac{b_{bp}}{2.0\times{10}^{-4}}$ (6)

Moreover, we have demonstrated that the association between the observed outcomes and actual Chl-*a* might also be impacted by diurnal variations, thus potentially introducing supplementary uncertainties in the calculations of daytime *p*CO_2_ and flux.

**CALIOP wind speed.** The input CALIOP variables were averaged 40 km along-track to match the AMSR-E footprint size. One limiting factor of space-based LiDAR measurements of ocean surface wind speed is the presence of thick clouds and aerosols. The matching window was set to 40 km along the AMSR-E track. CALIOP LiDAR measurements in 2008 (3692830 matched data) and ocean surface wind speed measurements from AMSR-E instruments were used for training the neural network CALIOP ocean surface wind speed approaches.

**Satellite and reanalyzed environmental datasets.** As shown in Table. 5, our predictors included biological, chemical, and physical variables commonly associated with variations in *p*CO_2_ (18-20): sea surface temperature, sea surface salinity, sea surface height, mixed-layer depth, Chl-*a*, and atmospheric CO_2_ mole fraction (xCO_2_). In addition to the predictors listed in this table, *p*CO_2_ climatology (21), normalized latitude (= sin(latitude×π/180º)) and longitude (= sin(longitude×π/180º) or = cos(longitude×π/180º)) were also used as predictors for the reconstruction. Two functions sin and cos for longitudes are used to preserve its periodic 0º to 360º behavior and to consider the difference of positions before and after the 0º longitude. Since the CALIOP data started in 2007, we used the merged satellite product GlobColour to reconstruct *p*CO_2_ from 1998 to 2006, which provides better spatial coverage than the product from a single satellite. To reduce the system difference in the two data between period when the GlobColour product was used, we conducted a sensitivity test of the developed approach between $d$*p*CO_2_(=${p\mathrm{CO}}_{2_{Globcolour}}-{p\mathrm{CO}}_{2_{CALIPSO}}$) and dChl-*a*(=${\mathrm{Chl}a}_{Globcolour}-{\mathrm{Chl}a}_{CALIPSO}$) since 2007. The test indicated the effect of using different products is not significant, with ($\partial d$*pCO*_2_/∂dChl*a*) being 0.4%±0.2%. Furthermore, the calculation of the C-Flux requires additional datasets, including atmospheric pressure, and 10 m wind speed at the sea surface, as shown in Table 5. The original data were distributed after interpolation on 1⁰ latitude by 1⁰ longitude cells. In reconstructing the diurnal *p*CO_2_ field and calculating the air-sea flux, we used all data with diurnal spatiotemporal resolution, including Chl*a*, wind speed, temperature, barometric pressure, and atmospheric CO_2_ concentration. In the context of diurnal research, they were treated as having the same spatial resolution, with analysis commencing in 2007.

Supplemental Methods

**FNN-LID model.** Ocean surface *p*CO_2_ is often constructed based on global ocean biogeochemical models and data reconstruction methods from satellite remote sensing environmental data, as illustrated in *SI Appendix*, Fig. S3*D*. In our study, we combined a novel feed-forward neural network methods with CALIOP LiDAR data to reconstruct the diurnal *p*CO_2_ from January 1998 to December 2020 on a monthly 1º × 1º resolution. Our approach involved a two-part method to establish nonlinear relationships between *p*CO_2_ and a set of independent environmental predictors. Further information on the feedforward neural network (FNN) training process can be found in Rumelhart et al. (1986) and Bishop (1995) (22, 23). In the first part, we derived a nonlinear and continuous relationship between climatology *p*CO_2_ and independent environmental predictors using FNN method. For the second part, the target data were provided by the gridded SOCATv2022 observational database.

Here, we utilized an advection-based interpolation method (21) to reconstruct the monthly *p*CO_2_ gridded climatology (1°×1°). In these two steps, the training datasets were expanded by combining data from the previous month and the next month to cluster the seasonal cycle and increase the dataset size in the second part. Furthermore, the dataset was divided into three sets: 50% for FNN training, 25% for evaluation, and 25% for model validation. The former 25% was excluded from the training process, which was used to monitor the performance of the FNN process. The remaining 25% was used for validation after training. To enhance the accuracy of the reconstruction and augment the sample quantity in sparse areas, as shown in Fig.13a, the model was trained separately for each month. Thus, there were 12 FNN models with five layers sharing a common architecture but trained on different datasets corresponding to each month. The validation results of this model are detailed in Section 2.1.

On this basis, we updated the input data to the diurnal CALIPSO surface sea Chl-*a*, diurnal xCO_2_ from ECMWF, and the diurnal SST product from MODIS for the period from 2007 to 2020. Hence, we reconstructed the day and night sea surface partial pressure of CO_2_ for the period from January 2007 to December 2020 at a monthly 1°×1° resolution.

**Calculation of the air-sea flux.** Global air-sea C-flux is commonly described through a bulk formula (24, 25):

$flux = k_{w}\times sol\times({p\mathrm{CO}}_{2_{water}}-{p\mathrm{CO}}_{2_{air}})$ (7)

where $k_{w}$ is the gas transfer velocity, $sol$ is the solubility of CO_2_ in seawater (in units of mol m^−3^ µatm^−1^), ${p\mathrm{CO}}_{2_{water}}$ is the partial pressure of sea surface CO_2_ (in µatm), and ${p\mathrm{CO}}_{2_{air}}$ (in units of µatm) represents the partial pressure of atmospheric CO_2_ in the marine boundary layer.

In this work, the $k_{w}$ is expressed as a function:

$k_{w}=0.259\times U_{10}\times{(\frac{Sc}{660})}^{-0.5}$ (8)

where $U_{10}$ is the wind speed 10 m above sea level, and $Sc$ denotes the Schmidt number calculated from the SST and SSS based on the equation proposed by Wanninkhof (2014). Although several equations have been proposed to parameterize $k_{w}$ as a function of $U_{10}$, we chose to use Eq. 8, as this equation has been widely used to estimate air-sea CO_2_ fluxes from regional to global scales with direct flux measurements (26, 27). To compute the diurnal air-sea carbon flux, we utilized all available data at diurnal resolution, encompassing wind speed, Chl-*a*, sea surface pressure, temperature, and xCO_2_ data. These diurnal datasets possessed identical spatial resolution as the monthly average results. All data used here can be sourced in Section 2. Throughout this study, flux was defined as positive when CO_2_ was released from the ocean to the atmosphere and negative when CO_2_ was absorbed by the ocean from the atmosphere.

**Statistics.**

The model robustness of the reconstructed *p*CO_2_ fields was evaluated using the gridded SOCAT data. The evaluation data were denoted as ${{pCO}_{2}^{Obs}}_{j}$ and were used in the following formulas to calculate mean absolute percentage error (MAPE), root-mean-squared error (RMSE), bias, and standard statistics included the coefficient of determination (*r*^2^).

$MAPE= \frac{100\%}{n}\sum_{i=1}^{n} \frac{\left| d_{j} \right|}{x_{obs}}$ (9)

$RMSE = \sqrt{\frac{\sum_{j=1}^{j=N} {(d_{j})}^{2}}{N}}$ (10)

$bias=\frac{\sum_{j=1}^{j=N} d_{j}}{N}$ (11)

where $d_{j}={{pCO}_{2}^{Est}}_{j}- {{pCO}_{2}^{Obs}}_{j}$, and N is the number of evaluation data. All these scores were computed for different coastal and open regions from the scale of grid cells to the global scale. The units of air–sea flux estimates were mmol C m^−2^ yr^−1^ for flux density, and this was converted to GtC yr^−1^ for an integral over a region or the global ocean.

**Uncertainty**

Uncertainty of the air-sea CO_2_ flux (γ) from equation (12) includes uncertainties in *p*CO_2_ ($\delta_{pCO2}$) and uncertainties in wind speed ($\delta_{wind}$).

$\frac{\Delta^{2}\gamma}{\gamma^{2}}= \frac{\Delta^{2}\delta_{pCO2}}{{\delta_{pCO2}}^{2}}+\frac{\Delta^{2}\delta_{wind}}{{\delta_{wind}}^{2}}$(12)

where $\gamma$ indicated the air-sea CO_2_ flux, $\delta_{pCO2}$ represented the *p*CO_2_ flux, $\delta_{wind}$ corresponds to the wind speed.

We have discerned three distinct sources of errors that collectively contribute to the overall uncertainty associated with *p*CO_2_ measurements. These error sources are rooted in the realms of measurement (M), representation (R), and prediction (P). By assuming the independence of these three error sources, we can derive the total uncertainty (E) for estimating *p*CO_2_. Mathematically, this uncertainty can be succinctly expressed as the square root of the sum of the squared uncertainties stemming from each of the three aforementioned error sources(28):

$E^{2}=M^{2}+R^{2}+P^{2}$(13)

The measurement error comprises potential biases from sampling and measurement, as well as random errors due to imprecision in the measurement system. Since *p*CO_2_ are measured against certified reference materials and undergo rigorous secondary quality control, we assume zero systematic error (bias) and consider the sampling error to be small. Therefore, the measurement error (M) is approximated by the precision of the employed measurement methodology (Dickson et al., 2007). The representation error (R) arises because our statistical model is developed on a grid that may have coarser temporal and spatial resolution compared to the natural variability of *p*CO_2_. Consequently, individual observations may not fully represent the monthly grid cell used for regression, leading to bias in the estimated mean relative to the true spatial and temporal mean. To address this, we use regions with sufficient observations or related parameters to approximate the uncertainty associated with representation error, assuming a globally averaged normal distribution with zero bias. Prediction error (P) uncertainty is determined from test scores during the evaluation of the statistical model against independent test data. These scores quantify the error in predicting data not used in the model training, including propagated uncertainty from predictor variables.

As for the RMSE value, we adopt a value of ±2 µatm for the uncertainty M associated with the measurement error of *p*CO_2_. This reflects the fact that we have used from SOCAT (flags A and B) with a precision better than that number and an accuracy of similar magnitude. For the uncertainty R associated with the representation error of *p*CO_2_, we estimated it based on a spatiotemporal gradient analysis. We compare the *p*CO_2_ in our regular grid, with the *p*CO_2_ binned to a grid with twice this resolution or higher spatiotemporal coverage. The spatial and temporal gradients are calculated separately, and we take the average of these two elements. Using this analysis, we estimate a representation error of our *p*CO_2_ estimates of 4.6 µatm. From the RMSE of our test data, we estimate an uncertainty P associated with the prediction error of *p*CO_2_ of 17.7 µatm, and specific RMSE values for different regions and years can be found in Table S3 and S4 of the Supplementary Information. The cluster-regression method estimates *p*CO_2_ with global near-zero biases and root mean squared errors of 18.4 µatm (approximately 4.6%). Taking the stability of the CALIOP depolarization ratio calibration and the CALIOP transient response function into account, we perturb the wind distributions by 10% (1). The cluster-regression method provides a total estimated uncertainty of 11% on the air-sea flux.

Fig. S1. (A-D) Map of mean seasonal amplitude in every 5 years during 2001–2020 in μatm. (E) The Map of the different latitudes and (F) the Long-term time-series changes of *p*CO_2_ during 2001–2020 in μatm.


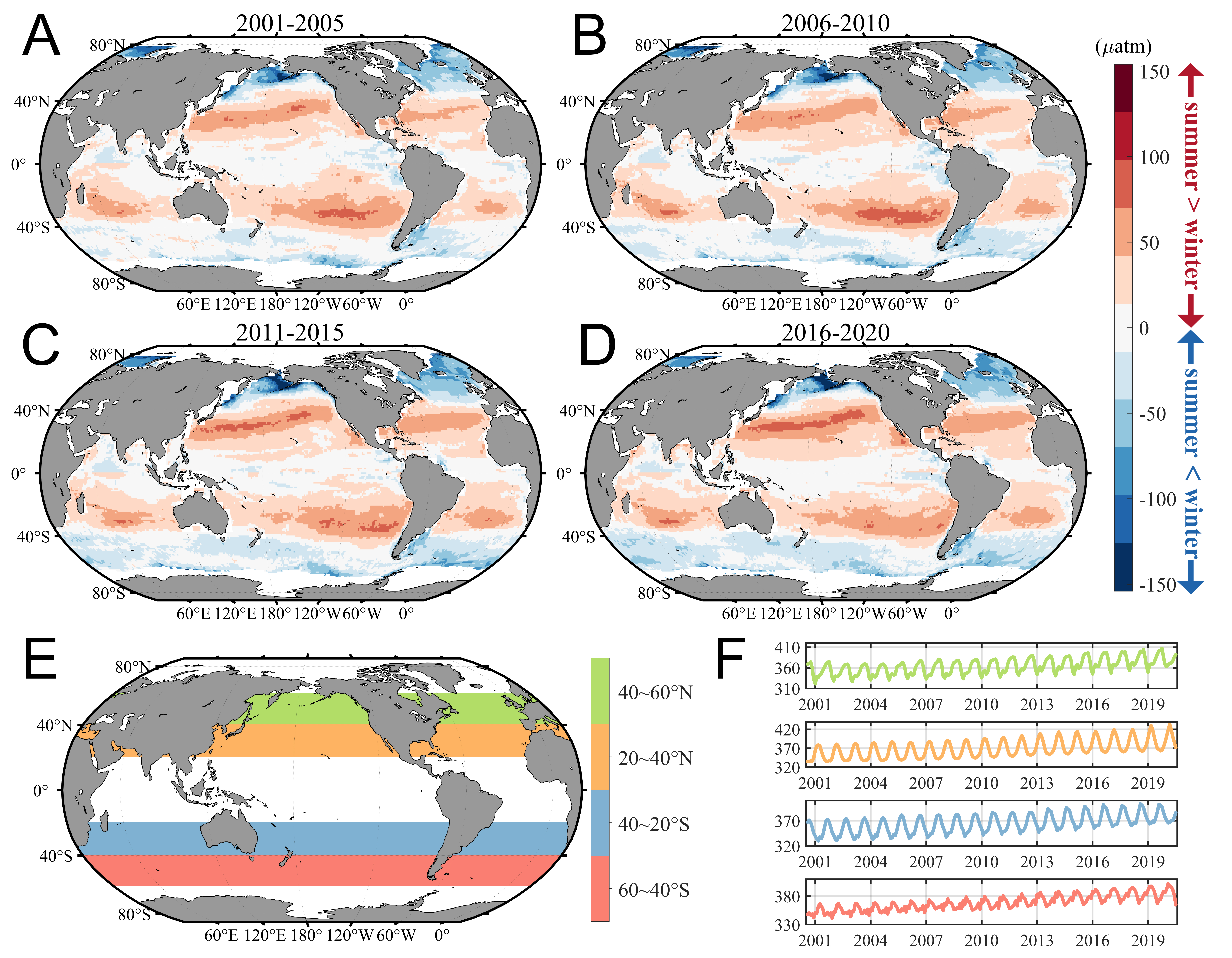


Fig. S2. (A) The scatterplot of collocated CALIOP and MODIS Chl-*a* with color presented for the number of measures; (B) the scatterplot of collocated CALIPSO and Bio-Argo Chl-*a*. All Chl-*a* values are in units of μg L^-1^. (C) The scatterplot of collocated CALIPSO and AMSR wind speed 10 m above sea level with color presented for the number of measures; (D) the scatterplot of collocated CALIPSO and buoy wind speed 10 m above sea level.


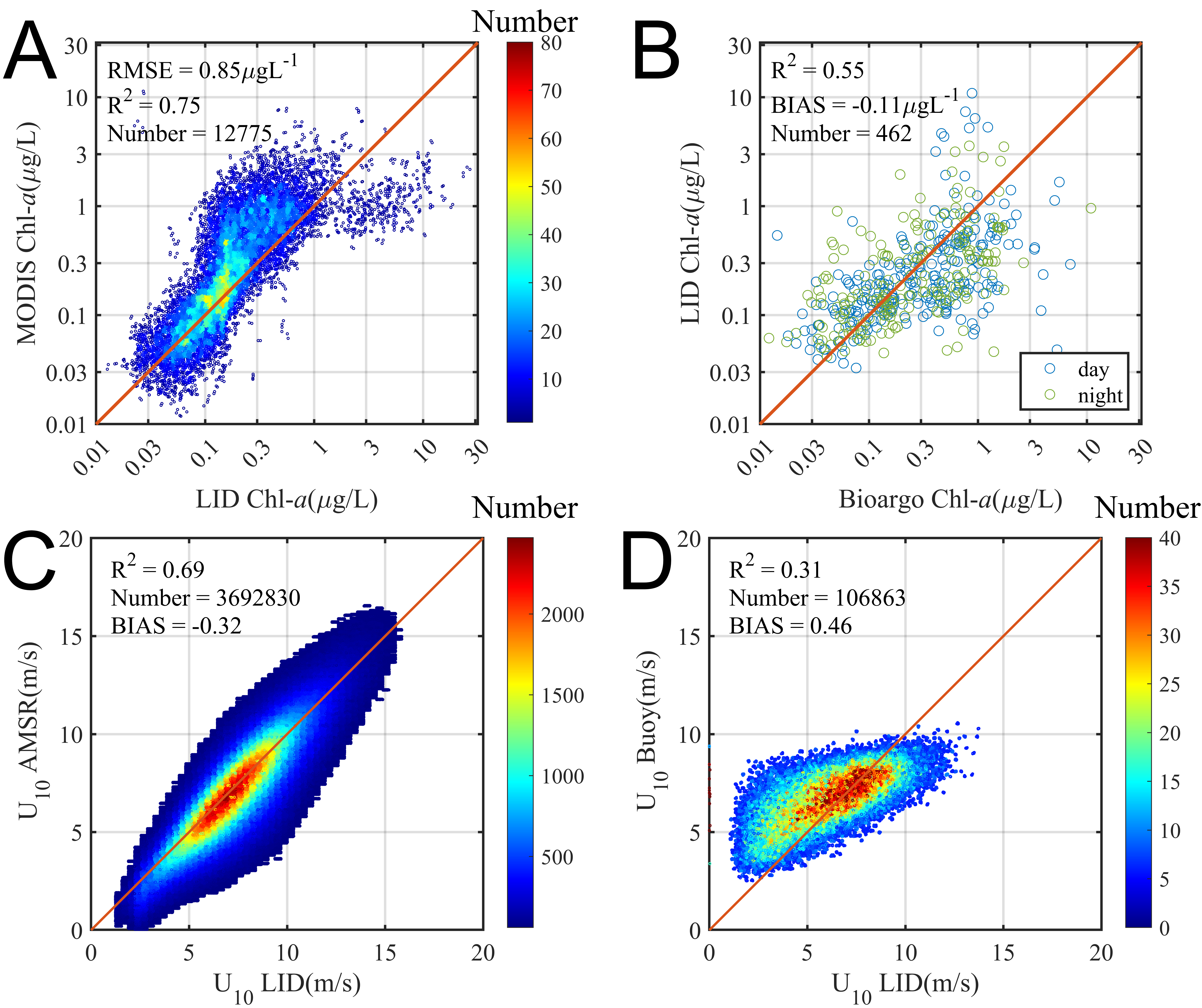


Fig. S3. (A) Map of the number of unique months with SOCATv2022 observations. (B) Map of Bio-Argo locations with red indicating day-matches and black indicating night-matches. (C) Map of buoy locations with wind data since 2010. (D) Illustration of the FFNN-LID model: A schematic description of the data flow for deriving diurnal surface ocean *p*CO_2_ products. Green arrows indicate the flow of the FNN approach, and yellow arrows indicate the estimation of production.


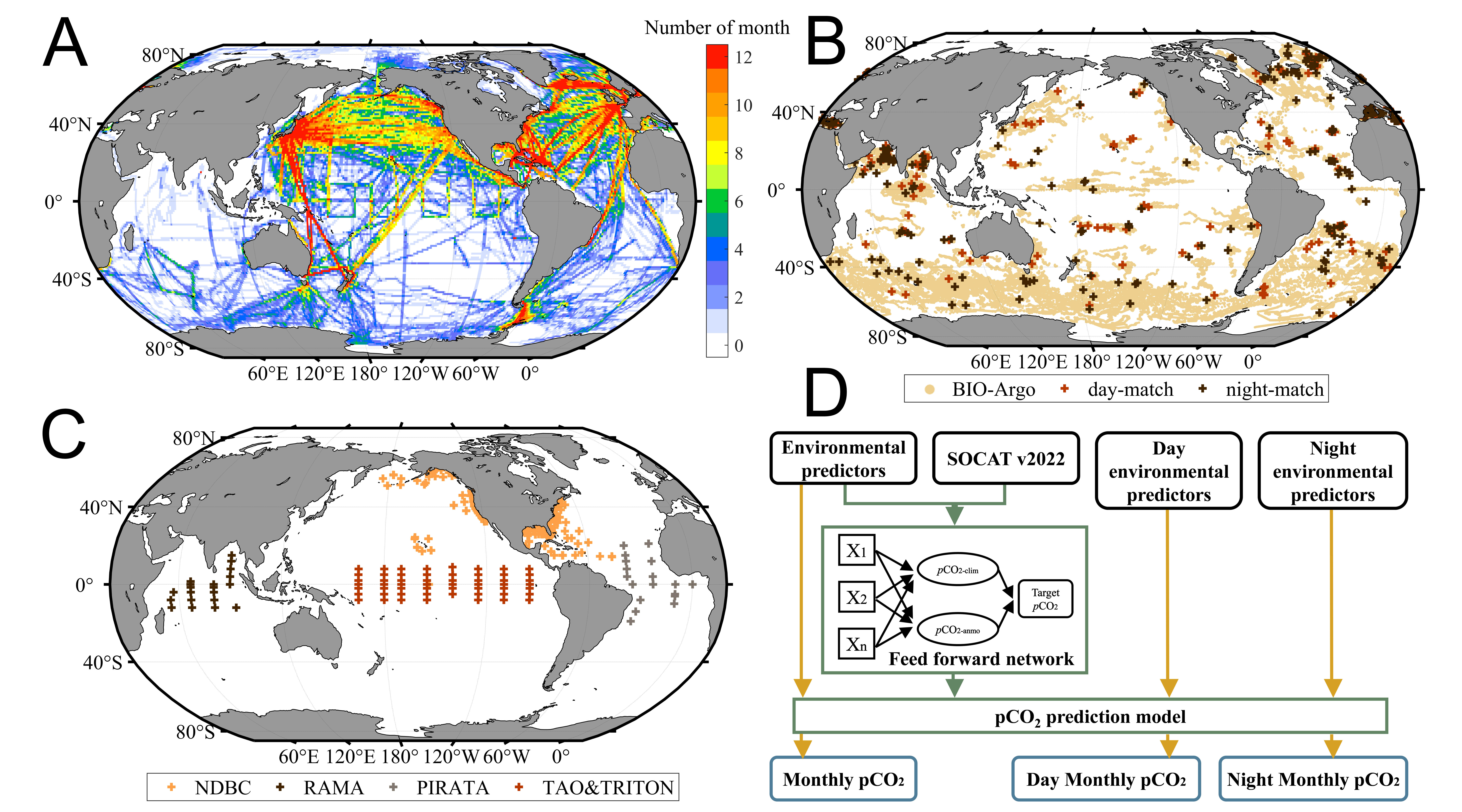


Table S1. Statistical measures of the comparison of the global FNN-LID estimates of *p*CO_2_ with the SOCATv2022 gridded data [Bakker et al., 2014], including the RMSE, *r*^2^, the bias between FNN-LID estimates and SOCATv2022 gridded data, and the number of grid cells.

| Year | RMSE  (μatm) | *r*^2^ | MAPE  (%) | BIAS  (μatm) | N |
| --- | --- | --- | --- | --- | --- |
| 1998–2020 | 17.74 | 0.79 | 3.16 | 0.13 | 250100 |
| 1998 | 16.77 | 0.75 | 3.35 | 1.41 | 5890 |
| 1999 | 19.71 | 0.76 | 3.59 | 1.61 | 4254 |
| 2000 | 21.08 | 0.77 | 3.93 | 1.23 | 5069 |
| 2001 | 20.29 | 0.79 | 3.73 | −0.93 | 5206 |
| 2002 | 17.56 | 0.73 | 3.23 | 1.18 | 7151 |
| 2003 | 15.26 | 0.78 | 2.95 | 0.31 | 7506 |
| 2004 | 15.18 | 0.81 | 2.91 | −0.21 | 8823 |
| 2005 | 16.30 | 0.80 | 3.05 | −0.97 | 10656 |
| 2006 | 16.74 | 0.82 | 3.33 | 0.34 | 13275 |
| 2007 | 17.55 | 0.77 | 3.40 | 0.10 | 12620 |
| 2008 | 15.80 | 0.84 | 3.11 | 0.08 | 11786 |
| 2009 | 17.82 | 0.75 | 3.33 | 1.09 | 11681 |
| 2010 | 16.28 | 0.76 | 2.98 | 0.48 | 12859 |
| 2011 | 18.17 | 0.77 | 3.25 | 0.34 | 13273 |
| 2012 | 18.40 | 0.76 | 3.15 | −0.02 | 13322 |
| 2013 | 17.15 | 0.77 | 3.13 | −0.30 | 11577 |
| 2014 | 18.43 | 0.81 | 3.42 | 1.16 | 13498 |
| 2015 | 20.35 | 0.73 | 3.20 | −1.11 | 13737 |
| 2016 | 16.02 | 0.80 | 2.85 | 0.07 | 15660 |
| 2017 | 17.45 | 0.81 | 3.18 | 0.97 | 15729 |
| 2018 | 17.75 | 0.77 | 3.11 | 0.53 | 13134 |
| 2019 | 19.09 | 0.77 | 3.15 | −0.84 | 12924 |
| 2020 | 17.27 | 0.76 | 3.35 | −2.02 | 10470 |

Table S2. Statistical measures of the comparison of the 26 regional oceans from FNN-LID estimates of *p*CO_2_ with the SOCATv2022 gridded data(1), including the RMSE, *r*^2^, the bias between FNN-LID estimates and SOCATv2022 gridded data (bias), and the number of grid cells.

| Name | RMSE  (μatm) | *r^2^* | MAPE  (%) | Bias  (μatm) | N |
| --- | --- | --- | --- | --- | --- |
| North Atlantic | 14.33 | 0.77 | 2.74 | 0.15 | 58405 |
| Coastal North Atlantic | 24.27 | 0.73 | 4.46 | −0.3 | 15826 |
| Equatorial Atlantic | 15.09 | 0.66 | 2.82 | −1.15 | 3970 |
| Coastal Equatorial Atlantic | 31.06 | 0.44 | 4.75 | 1.52 | 460 |
| South Atlantic | 13.73 | 0.77 | 2.84 | −0.31 | 5337 |
| Coastal South Atlantic | 25.99 | 0.69 | 5.33 | −0.43 | 1193 |
| North Pacific | 15.68 | 0.81 | 2.79 | 0.36 | 75296 |
| Coastal North Pacific | 39.52 | 0.61 | 6.52 | −1.02 | 4557 |
| Equatorial Pacific | 15.94 | 0.84 | 2.71 | 0.57 | 21700 |
| Coastal Equatorial Pacific | 19.47 | 0.51 | 3.33 | 5.52 | 500 |
| South Pacific | 10.52 | 0.79 | 2.17 | 0.81 | 15956 |
| Coastal South Pacific | 17.9 | 0.5 | 3.45 | −1.26 | 2497 |
| North Indian | 14.45 | 0.66 | 2.91 | −1.52 | 122 |
| Coastal North Indian | 21.33 | - | 5.08 | 20.97 | 2 |
| Equatorial Indian | 10.65 | 0.61 | 2.1 | 1.19 | 400 |
| Coastal Equatorial Indian | 17.18 | - | 3.76 | −4.76 | 11 |
| South Indian | 12.31 | 0.74 | 2.57 | −0.28 | 6698 |
| Coastal South Indian | 17.28 | 0.54 | 3.64 | −0.68 | 926 |
| Antarctic | 21.64 | 0.68 | 4.2 | −0.14 | 24677 |
| Arctic | 25.3 | 0.74 | 5.8 | −0.52 | 11567 |

Table S3. The air-sea C-flux of the 26 oceans from the FNN-LID estimates, including the location and dimension.

|  | Latitude  boundaries | Dimension  ×10^5^ km^2^ | *p*CO_2_  (μatm) | Carbon sink  Pg C yr^-1^ |
| --- | --- | --- | --- | --- |
| North Atlantic | 10-66°N | 293.35 | 361.92 | -0.3930 |
| Coastal North Atlantic | 10-66°N | 36.47 | 363.05 | -0.0600 |
| Equatorial Atlantic | 10°S-10°N | 112.71 | 388.75 | 0.0506 |
| Coastal Equatorial Atlantic | 10°S-10°N | 6.28 | 380.96 | 0.0015 |
| South Atlantic | 50-10°S | 252.58 | 372.32 | -0.2860 |
| Coastal South Atlantic | 50-10°S | 13.07 | 361.93 | -0.0209 |
| North Pacific | 10-65°N | 525.23 | 368.14 | -0.4424 |
| Coastal North Pacific | 10-61°N | 24.17 | 361.73 | -0.0214 |
| Equatorial Pacific | 10°S-10°N | 370.15 | 410.02 | 0.3977 |
| Coastal Equatorial Pacific | 10°S-10°N | 16.30 | 385.66 | 0.0032 |
| South Pacific | 50-10°S | 539.84 | 365.56 | -0.4863 |
| Coastal South Pacific | 50-10°S | 14.25 | 364.55 | -0.0102 |
| North Indian | 10-25°N | 34.55 | 404.88 | 0.0393 |
| Coastal North Indian | 10-25°N | 7.40 | 402.56 | 0.0061 |
| Equatorial Indian | 10°S-10°N | 132.41 | 395.18 | 0.0747 |
| Coastal Equatorial Indian | 10°S-10°N | 10.03 | 396.26 | 0.0082 |
| South Indian | 50-10°S | 367.22 | 360.92 | -0.5761 |
| Coastal South Indian | 50-10°S | 13.70 | 362.27 | -0.0104 |
| Antarctic | 90-50°S | 344.29 | 367.77 | -0.0990 |
| Arctic | 65-90°N | 40.43 | 324.55 | -0.4030 |
| Sulu Sea | 6-10°N | 2.45 | 375.08 | 0.0001 |

Table S4. The CALIOP data used in retrieving sea surface wind speed.

|  | Components | Wavelength | Polarization |
| --- | --- | --- | --- |
| 1 | Ocean surface and subsurface lidar backscatter | 532 nm | Total |
| 2 | Column integrated atmospheric lidar backscatter | 532 nm | Total |
| 3 | Ocean surface and subsurface lidar backscatter | 532 nm | Perpendicularly |
| 4 | Column integrated atmospheric lidar backscatter | 532 nm | Perpendicular |
| 5 | Ocean surface and subsurface lidar backscatter | 1,064 nm | - |
| 6 | Column integrated atmospheric lidar backscatter | 1,064 nm | - |
| 7 | Latitude | - | - |

Table S5. Details of satellite and reanalyzed input fields.

| Satellite and reanalyzed environmental dataset for reconstructing surface ocean *p*CO_2_ | | | |
| --- | --- | --- | --- |
| Components | Dataset | Time scale | Website |
| Sea surface temperature | MODIS | Monthly/diurnal | https://resources.marine.copernicus.eu/products |
| Sea surface salinity | CMEMS | Monthly | https://resources.marine.copernicus.eu/products |
| Sea surface height | CMEMS | Monthly | https://resources.marine.copernicus.eu/products |
| Mixed layer depth | Menemenlis et al., 2008 | Monthly | https://www.ecco-group.org/products.htm |
| Chl-*a* | GlobColour CALIPSO | Monthly Monthly/diurnal | https://www.globcolour.info/products_description.html |
| Atmospheric CO_2_ mole fraction | ECMWF | Monthly/diurnal | https://ads.atmosphere.copernicus.eu |
| Climatology *p*CO_2_ | Takahashi et al. (2009) | Monthly | - |
| Satellite and reanalyzed environmental dataset for reconstruction of air-sea C-flux | | | |
| Sea level pressure 10 m wind speed | CALIPSO | Monthly/diurnal | - |
| Total pressure | ECMWF | Monthly/diurnal | https://ads.atmosphere.copernicus.eu |

**SI References**

1. D. C. Bakker *et al.*, A multi-decade record of high-quality fCO 2 data in version 3 of the Surface Ocean CO 2 Atlas (SOCAT). *Earth System Science Data* **8**, 383-413 (2016).

2. A. Körtzinger, Methods of Seawater Analysis, chap. *Determination of carbon dioxide partial pressure (pCO*_2_*)*, 149-158 (1999).

3. H. R. Weiss, Control of myocardial oxygenation—effect of atrial pacing. *Microvascular research* **8**, 362-376 (1974).

4. E. Kalnay *et al.*, The NCEP/NCAR 40-year reanalysis project. *Bulletin of the American meteorological Society* **77**, 437-472 (1996).

5. K. S. Johnson *et al.*, Observing biogeochemical cycles at global scales with profiling floats and gliders: prospects for a global array. *Oceanography* **22**, 216-225 (2009).

6. H. Claustre, Bio-Optical Sensors on Argo Floats. (2011).

7. M. J. McPhaden, The tropical atmosphere ocean array is completed. *Bulletin of the American Meteorological Society* **76**, 739-741 (1995).

8. Y. KURODA, Y. AMITANI, TRITON: New Ocean and Atmosphere Observing Buoy Network for Monitoring ENSO. *海の研究* **10**, 157-172 (2001).

9. J. Servain *et al.*, A pilot research moored array in the tropical Atlantic (PIRATA). *Bulletin of the American Meteorological Society* **79**, 2019-2032 (1998).

10. M. J. Mcphaden *et al.*, RAMA: the research moored array for African–Asian–Australian monsoon analysis and prediction. *Bulletin of the American Meteorological Society* **90**, 459-480 (2009).

11. D. B. Gilhousen, A field evaluation of NDBC moored buoy winds. *Journal of Atmospheric and Oceanic Technology* **4**, 94-104 (1987).

12. W. T. Liu, W. Tang (1996) Equivalent neutral wind.

13. X. Lu, Y. Hu, M. Vaughan, S. Rodier, A. Omar, New attenuated backscatter profile by removing the CALIOP receiver's transient response. *Journal of Quantitative Spectroscopy and Radiative Transfer*, 107244 (2020).

14. J. Li *et al.*, A new method for retrieval of the extinction coefficient of water clouds by using the tail of the CALIOP signal. *Atmospheric Chemistry and Physics,11,6(2011-03-29)* **10**, 2903-2916 (2011).

15. M. J. Behrenfeld *et al.*, Space‐based lidar measurements of global ocean carbon stocks. *Geophysical Research Letters* **40**, 4355-4360 (2013).

16. K. M. Bisson, E. Boss, P. J. Werdell, A. Ibrahim, M. J. Behrenfeld, Particulate Backscattering in the Global Ocean: A Comparison of Independent Assessments. *Geophysical Research Letters* **48** (2021).

17. X. Lu, Y. Hu, C. Trepte, S. Zeng, J. H. Churnside, Ocean subsurface studies with the CALIPSO spaceborne lidar. *Journal of Geophysical Research: Oceans* **119**, 4305-4317 (2014).

18. P. Landschützer *et al.*, A neural network-based estimate of the seasonal to inter-annual variability of the Atlantic Ocean carbon sink. *Biogeosciences* **10**, 7793-7815 (2013).

19. A. Denvil-Sommer, M. Gehlen, M. Vrac, C. Mejia, ffnn-lsce: a two-step neural network model for the reconstruction of surface ocean pco 2 over the global ocean. *Geoscientific Model Development* (2019).

20. L. Gregor, A. D. Lebehot, S. Kok, P. M. Scheel Monteiro, A comparative assessment of the uncertainties of global surface ocean CO 2 estimates using a machine-learning ensemble (CSIR-ML6 version 2019a)–have we hit the wall? *Geoscientific Model Development* **12**, 5113-5136 (2019).

21. T. Takahashi *et al.*, Climatological mean and decadal change in surface ocean *p*CO_2_, and net sea–air CO_2_ flux over the global oceans. *Deep Sea Research Part II: Topical Studies in Oceanography* **56**, 554-577 (2009).

22. D. E. Rumelhart, G. E. Hinton, R. J. Williams, Learning representations by back-propagating errors. *nature* **323**, 533-536 (1986).

23. C. M. Bishop, *Neural networks for pattern recognition* (Oxford university press, 1995).

24. C. S. Garbe *et al.*, "Transfer across the air-sea interface" in Ocean-atmosphere interactions of gases and particles. (Springer, Berlin, Heidelberg, 2014), pp. 55-112.

25. R. Wanninkhof, Relationship between wind speed and gas exchange over the ocean revisited. *Limnology and Oceanography: Methods* **12**, 351-362 (2014).

26. C. Rödenbeck *et al.*, Data-based estimates of the ocean carbon sink variability–first results of the Surface Ocean pCO 2 Mapping intercomparison (SOCOM). *Biogeosciences* **12**, 7251-7278 (2015).

27. J. Prytherch *et al.*, Direct determination of the air‐sea CO_2_ gas transfer velocity in Arctic sea ice regions. *Geophysical Research Letters* **44**, 3770-3778 (2017).

28. L. Gregor, N. Gruber, OceanSODA-ETHZ: a global gridded data set of the surface ocean carbonate system for seasonal to decadal studies of ocean acidification. *Earth System Science Data* **13**, 777-808 (2021).
